# Novel 5′-Norcarbocyclic Pyrimidine Derivatives as Antibacterial Agents

**DOI:** 10.3390/molecules23123069

**Published:** 2018-11-23

**Authors:** Anastasia L. Khandazhinskaya, Liudmila A. Alexandrova, Elena S. Matyugina, Pavel N. Solyev, Olga V. Efremenkova, Karen W. Buckheit, Maggie Wilkinson, Robert W. Buckheit, Larisa N. Chernousova, Tatiana G. Smirnova, Sofya N. Andreevskaya, Olga G. Leonova, Vladimir I. Popenko, Sergey N. Kochetkov, Katherine L. Seley-Radtke

**Affiliations:** 1 Engelhardt Institute of Molecular Biology of the Russian Academy of Sciences, 32 Vavilov St., Moscow 119991, Russia; khandazhinskaya@bk.ru (A.L.K.); ala2004_07@mail.ru (L.A.A.); matyugina@gmail.com (E.S.M.); solyev@gmail.com (P.N.S.); varjag@aport2000.ru (O.G.L.); popenko@eimb.ru (V.I.P.); kochet@eimb.ru (S.N.K.); 2 Gause Institute of New Antibiotics, 11 Bol’shaya Pirogovskaya St., Moscow 119021, Russia; ovefr@yandex.ru; 3 ImQuest BioSciences, 7340 Executive Way Suite R, Frederick, MD 21704, USA; kbuckheit@imquestbio.com (K.W.B.); mwilkinson@imquestbio.com (M.W.); rbuckheit@imquestbio.com (R.W.B.Jr.); 4 Central Tuberculosis Research Institute, 2 Yauzskaya Alley, Moscow 107564, Russia; lchernousova@mail.ru (L.N.C.); s_tatka@mail.ru (T.G.S.); andsofia@mail.ru (S.N.A.); 5 Department of Chemistry & Biochemistry, University of Maryland, 1000 Hilltop Circle, Baltimore, MD 21250, USA

**Keywords:** 5′-norcarbocyclic nucleosides, *Mycobacterium tuberculosis*, antibacterial activity, uracil derivatives

## Abstract

A series of novel 5′-norcarbocyclic derivatives of 5-alkoxymethyl or 5-alkyltriazolyl-methyl uracil were synthesized and the activity of the compounds evaluated against both Gram-positive and Gram-negative bacteria. The growth of *Mycobacterium smegmatis* was completely inhibited by the most active compounds at a MIC_99_ of 67 μg/mL (mc^2^155) and a MIC_99_ of 6.7–67 μg/mL (VKPM Ac 1339). Several compounds also showed the ability to inhibit the growth of attenuated strains of *Mycobacterium tuberculosis* ATCC 25177 (MIC_99_ 28–61 μg/mL) and *Mycobacterium bovis* ATCC 35737 (MIC_99_ 50–60 μg/mL), as well as two virulent strains of *M. tuberculosis*; a laboratory strain H37Rv (MIC_99_ 20–50 μg/mL) and a clinical strain with multiple drug resistance MS-115 (MIC_99_ 20–50 μg/mL). Transmission electron microscopy (TEM) evaluation of *M. tuberculosis* H37Rv bacterial cells treated with one of the compounds demonstrated destruction of the bacterial cell wall, suggesting that the mechanism of action for these compounds may be related to their interactions with bacteria cell walls.

## 1. Introduction

**Figure 1:**
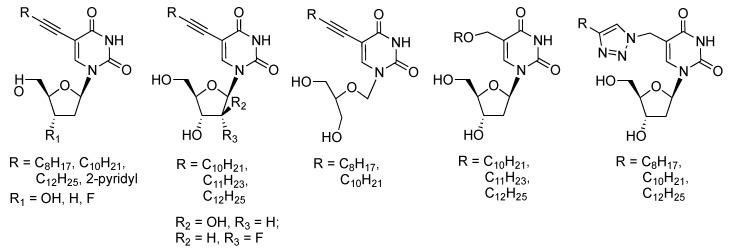
Previously reported anti-*M. tuberculosis* nucleosides.

Antibiotic resistance has emerged for nearly all currently used therapeutics for many diseases, including tuberculosis (TB) [1,2]. Despite this, the development of new classes of antibiotics has lagged far behind the field’s growing need for drugs that can act on new targets and remain active against resistant strains of pathogens. Nucleoside derivatives have historically served as potent antiviral and anticancer agents [3], but it was not until 2000 that their anti-TB activity was reported [4]. Recently, several reports of modified nucleosides showing activity against *Mycobacterium tuberculosis (M. tuberculosis)*, *Mycobacterium bovis (M. bovis)* and *Mycobacterium avium (M. avium)* were published [4,5,6,7,8,9,10,11,12,13,14,15,16,17,18]. Among them, the best activity was observed for several uridine analogues (MIC_90_ 1 to 10 µg/mL) possessing 5-alkynyl (decynyl, dodecynyl, tetradecynyl, pyridylethynyl, etc.) substituents as well as modified sugar moieties (Figure 1). In addition, several 2′-deoxypyrimidine derivatives with extended 5-alkyloxymethyl or 5-alkyltriazolylmethyl substituents (5-dodecyloxy-methyl-2′-deoxyuridine, 5-decyltriazolylmethyl-2′-deoxyuridine, 5-dodecyltriazolylmethyl-2′-deoxycytidine, etc.) were shown to inhibit in vitro growth of both laboratory H37Rv and clinical multiple drug resistant (MDR) MS-115 *M. tuberculosis* strains with MIC_99_ values ~10 µg/mL (Figure 1) [5].

In an effort to explore additional sugar modifications, a series of carbocyclic nucleoside analogues with the same modifications were designed and synthesized. Because it had already been shown that uracil analogues with short alkyne or halogen substituents at the C-5 position failed to inhibit growth of *M. tuberculosis* [4,17,18], we chose to synthesize longer substituents. Several showed promise, exhibiting activity below 100 μg/mL. Their synthesis and subsequent biological investigations are discussed below.

## 2. Results and Discussion

Carbocyclic nucleosides are analogues of natural nucleosides in which the oxygen atom of the furanose ring is replaced by a methylene group. In addition, several carbocyclic nucleosides, for example, aristeromycin and neplanocin A, have been found in Nature [19,20,21,22]. Nucleosides of this type are recognized by many receptors and enzymes due to their structural similarity to the natural nucleosides, however they display increased stability towards phosphorylase- and hydrolase-induced cleavage of the pseudo-glycosidic bond. Moreover, they possess a wide spectrum of biological activity, particularly, antiviral and anticancer properties [19,23,24,25,26,27,28,29,30,31,32,33]. Unfortunately, in some cases they have exhibited significant toxicity as a result of their conversion to triphosphate forms, which closely resemble natural nucleoside triphosphates (NTP), thus resulting in unwanted recognition by ATP metabolizing enzymes [34,35,36].

**Figure 2:**
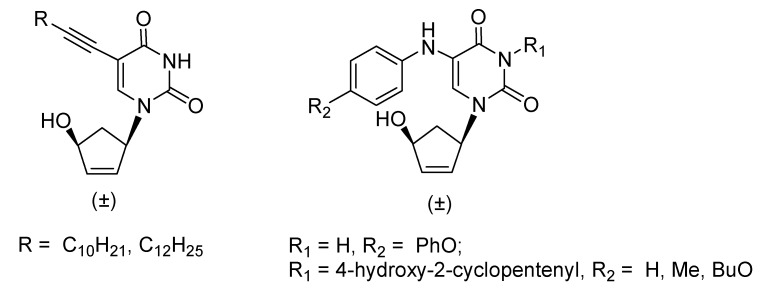
Examples of anti-Mtb 5′-norcarbocyclic nucleosides.

To overcome the cellular cytotoxicity of carbocyclic nucleosides that inhibit their target enzymes without preliminary phosphorylation (for example, aristeromycin and neplanocin A) [35], the 5′-norcarbocyclic nucleosides were designed. The switch of a secondary hydroxyl group for the primary hydroxyl group at C-4′ led to a lack of recognition by cellular kinases and as a result, they were less toxic [36,37,38,39,40,41,42,43,44,45,46]. Recently, we have shown that 5′-norcarbocyclic nucleosides with a 5-substituted uracil base (Figure 2) can act as promising anti-TB agents (MIC_99_ 5–10 µg/mL on both laboratory strain *M. tuberculosis* H37Rv and clinical isolate *M. tuberculosis* MDR MS-115) [47,48]. Despite our best efforts, however, their mechanism of action remains elusive.

In that regard, the thymidylate kinases of *M. tuberculosis* were evaluated as possible enzymatic targets of 1-(4′-hydroxy-2′-cyclopenten1′-yl)-5-(phenylamino)uracils and 1,3-di-(4′-hydroxy-2′-cyclopenten-1′-yl)-5-(phenylamino)uracils. No inhibition was found up to 200 µM [47]. Another possible mechanism of action that was considered was the inhibition of *M. tuberculosis* thymidylate synthases, thus both the classical (ThyA) and flavin dependent thymidylate synthase (ThyX)A were evaluated [46]. All of the derivatives lacked activity against the ThyA and only one inhibited ThyX at a concentration of 8.32 µM. Thus it was assumed that the anti-TB activity of 5-alkoxymethyl or 5-alkyltriazolylmethyl 2′-deoxyuridine analogues could not be explained by inhibition of thymidine kinases.

**Figure 3:**
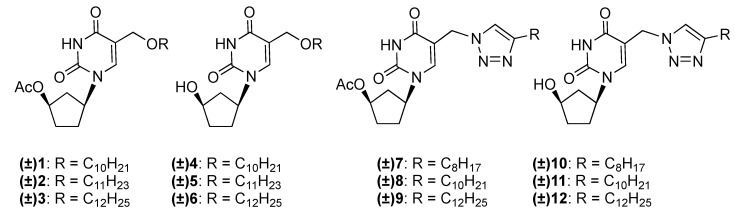
Target compounds.

In an effort to further explore the mechanism of action for these compounds, we have now designed a new hybrid scaffold possessing both modifications. Herein, we describe the synthesis and preliminary antibacterial activity for a series of new 5′-norcarbocyclic pyrimidine derivatives containing extended 5-alkyloxymethyl or 5-alkyltriazolylmethyl residues (Figure 3).

### 2.1. Chemistry

**Scheme 1:**
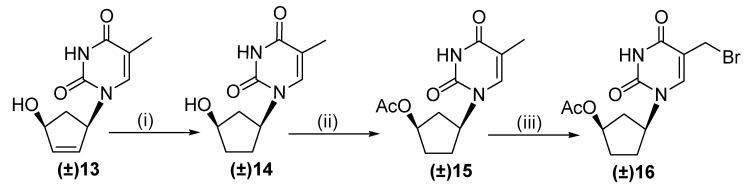
(i) Pd/C, H_2_, MeOH; (ii) Ac_2_O, Py; (iii) Br_2_, DCE, *hv*, 83 °C.

The key intermediate needed for the synthesis of both scaffolds for the new 5-modified 5′-norcarbocyclic analogues is racemic 1-(4′-acetoxycyclopent-1′-yl)-5-(bromomethyl)-uracil (**16**). As shown in Scheme 1, **16** was synthesized in three steps starting from 1-(4′-hydroxy-2′-cyclopenten-1′-yl)-thymine (**13**), which was obtained by the Trost procedure [49] using the coupling of epoxy-cyclopentene with thymine. Hydrogenation of **13** in the presence of palladium on carbon gave 1-(4′-hydroxycyclopent-1′-yl)-thymine (**14**) which was protected to provide 1-(4′-acetoxycyclopent-1′-yl)thymine (**15**). Following free radical bromination by the Barwolff and Langen procedure [50], key intermediate **16** was obtained.

**Scheme 2:**
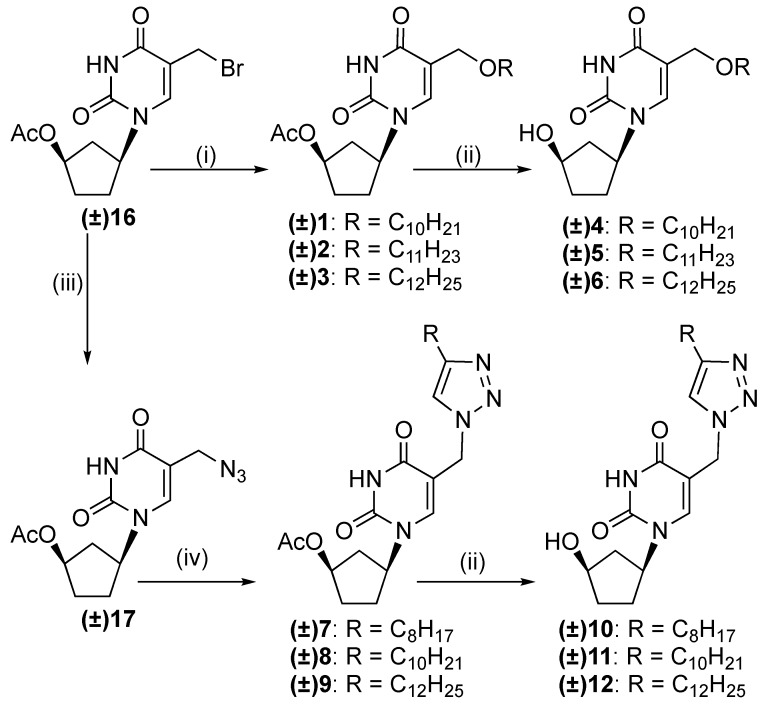
(i) ROH; (ii) K_2_CO_3_, MeOH; (iii) NaN_3_, DMF, 40 °C; (iv) RC≡CH, CuSO_4_·5H_2_O, sodium ascorbate, CH_2_Cl_2_/H_2_O (1:1).

The desired 5-alkyloxymethyl substituted analogues **1**–**3** were then prepared by coupling with the corresponding 1-alcohol [51] and subsequent deprotection of the 4’-hydroxy group to provide targets **4**–**6** (Scheme 2).

The 5-alkyltriazolylmethyl derivatives **7**–**12** were synthesized by the Lee method (Scheme 2) [52]. Azidation of 1-(4′-acetoxycyclopent-1′-yl)-5-(bromomethyl)uracil (**16**) to the corresponding 5-azidomethyl **17** was followed by 1,3-dipolarcycloaddition with various 1-acetylenes in a biphasic system of dichloromethane-water under catalytic conditions using Cu(I) obtained in situ from copper sulphate and sodium ascorbate to give **7**–**9**. After deprotection of the 4′-hydroxyl group of **7**–**9**, the desired 5-alkyltriazolylmethyl derivatives **10**–**12** were obtained in good yields (84–92%). It should be noted that the synthetic route from **16** to **1**–**12** doesn’t change the configuration of the chiral centers, i.e., C-1′ and C-4′. Thus, because compound 16 is racemic all of the target compounds (**1**–**12**) were obtained as racemic mixtures. Moreover, following synthesis of the individual isomers of the compound with the 5-dodecynyl substituent (Figure 2) no significant difference in the activity was found (see [47]), thus we chose not to synthesize the individual enantiomers of the other target compounds.

### 2.2. Biological Evaluation and Structure-Activity Relationship Studies

In order to determine the lead compounds prior to evaluation against the virulent strains of *M. tuberculosis*, the twelve compounds were initially evaluated for inhibition of an attenuated strain of *M. tuberculosis* ATCC 25177 (H37Ra), which is closely related to a virulent laboratory strain H37Rv, as well as the *M. bovis* ATCC strain 35737 in an in vitro assay (Table 1). When evaluated against *M. tuberculosis* ATCC strain 25177, the 5-alkyloxymethyl substituted analogues **1**–**3** had MIC_99_ values of 409, 423 and 54 µg/mL, respectively. Deprotection of the 4′-hydroxygroup resulted in targets **4**–**6** having MIC values of 183, 191 and 198 µg/mL. The 5-alkyltriazolylmethyl derivatives **7**–**12** had increased activities ranging from 29 µg/mL to 98 µg/mL.

Compounds **1**–**12** have similar activities against *M. bovis* ATCC strain 35737 with MICs either the same or within 2-fold of the MICs determined for *M. tuberculosis*. The exception was compounds **9** and **12**, which exhibited an 8-fold decrease in activity against *M. bovis* relative to *M. tuberculosis*. Thus, the best antimycobacterial activity against both strains among the 5-alkyloxymethyl derivatives **1**–**6** was seen for compound **3** with the 4′-acetyl group and the methyloxydodecyl substituent at the 5-position of the uracil moiety. In the series of 5-alkyltriazolylmethyl derivatives **7**–**12**, the 4′-acetylated analogues **7** (5-octyltriazolylmethyl) and 8-(5-decyltriazolylmethyl) were observed to have a significant inhibitory effect. DMSO (5%, equivalent to the same amount as would be present in the final solutions of the tested compounds) was evaluated in parallel and was determined to be non-toxic to all of the organisms.

The antibacterial activity of the synthesized compounds was also evaluated against Gram-negative bacteria including *Pseudomonas aeruginosa* (*P. aeruginosa*) ATCC strain 27853 and *Escherichia coli* (*E. coli*) ATCC strain 25922, Gram-positive bacteria including *Bacillus subtilis (B. subtilis)* ATCC strain 6633, *Staphylococcus aureus* (*S. aureus*) INA 00761 (MRSA) and *Leuconostoc mesenteroides* (*L. mesenteroides*) VKPM B-4177. *Mycobacterium smegmatis* (*M. smegmatis*) mc^2^155 and *M. smegmatis* VKPM Ac 1339, were also used in the primary screening for potential antibiotic activity. Most of the bacteria were not sensitive to the tested compounds. The only exception was against *M. smegmatis*, whose growth was completely inhibited by compounds **3**, **6**, **8** and **11** at a concentration of 6.7–67 μg/mL (Table 1). The MIC_99_ of the control antibiotics rifampicin and isoniazid were 10 and 1 μg/mL for *M. smegmatis* VKPM Ac 1339, and > 100 and 64 μg/mL for *M. smegmatis* mc^2^155, respectively. Interestingly, the tested compounds demonstrated selective activity only on mycobacteria, but not on other gram-positive and gram-negative bacteria (data not shown), thus could represent a specific interaction that will be pursued in subsequent studies. Three compounds (**3**, **7** and **8**) were selected as leads against the attenuated strain ATCC 25177 and subjected to additional antimycobacterial screening. The ability of the new 5′-norcarbocyclic nucleosides to influence the growth of two virulent strains of *M. tuberculosis*, including the laboratory strain *M. tuberculosis* H37Rv and the clinical strain *M. tuberculosis* MS-115 with multiple drug resistance were assessed in vitro. The antimycobacterial effect was studied by measuring the growth dynamics of strains of *M. tuberculosis* in the enriched medium Middlebrook 7H9 in the automated growth registration system BACTEC MGIT 960. Different concentrations of the compounds were compared to the growth of strains on media not containing preparations and media containing control preparations at critical concentrations (rifampicin 1 μg/mL, isoniazid 0.1 μg/mL and levofloxacin 1.5 μg/mL). Each of the concentrations of the tested compounds as well as control samples were done in triplicate. In all positive samples, the culture of mycobacteria of the tuberculosis complex was confirmed to grow and there was no contamination with nonspecific microflora. When exposed to control preparations at critical concentrations, the growth of the sensitive culture was not fixed, and the growth parameters of the MDR strain when incubated with isoniazid and rifampicin did not differ from the control without the drug.

As shown in Table 2, the MIC_99_ against the laboratory sensitive strain *M. tuberculosis* H37Rv were for compounds **3** (20 μg/mL), **7** (50 μg/mL) and **8** (50 μg/mL). Against *M. tuberculosis* MS-115, the MIC_99_ values for compounds **3**, **7**, and **8** were 20 μg/ mL, 20 μg/mL, and 50 μg/mL, respectively. It should be noted that unlike the previously reported 5-alkynyl substituted compounds, the 5-alkoxymethyl and 5-alkyl-triazolylmethyl derivatives proved to be quite toxic to the monocytic cells. The cytotoxicity of compounds **3**, **7** and **8** to the U-937 laboratory adapted monocytic cell line was similar to or greater than the activity of the compounds against the mycobacteria with TC_50_ values 20.6 μg/mL, 23.7 μg/mL and 15.1 μg/mL, respectively. Nonoxynol-9 was also evaluated in parallel as a positive control and was toxic at the expected concentration. So while it is clear these compounds are too toxic to be regarded as drug candidates, they may be useful as a tool for better understanding the mechanism of antimicobacterial action for this class of compounds.

In contrast, the observed biological activity of these new derivatives of 5-substituted uracils are in good correlation with previously reported data on the inhibition of M.tb growth by nucleoside analogues containing 5-substituted pyrimidine bases and different sugars, as well as their corresponding acyclic, or carbocyclic analogues [4,5,15,16,17,18,53] As a result, it appears these compounds may have the same mechanism of antimicrobial action (likely cell wall disruption) and that it depends primarily on the length and structure of the substituent at C-5 of the base moiety. Thus this new data has added to the growing examples of similar compounds and their mechanism of action for inhibitors of this type. In an attempt to further support this hypothesis some additional experiments using method of transmission electron microscopy were carried out.

**Figure 4:**
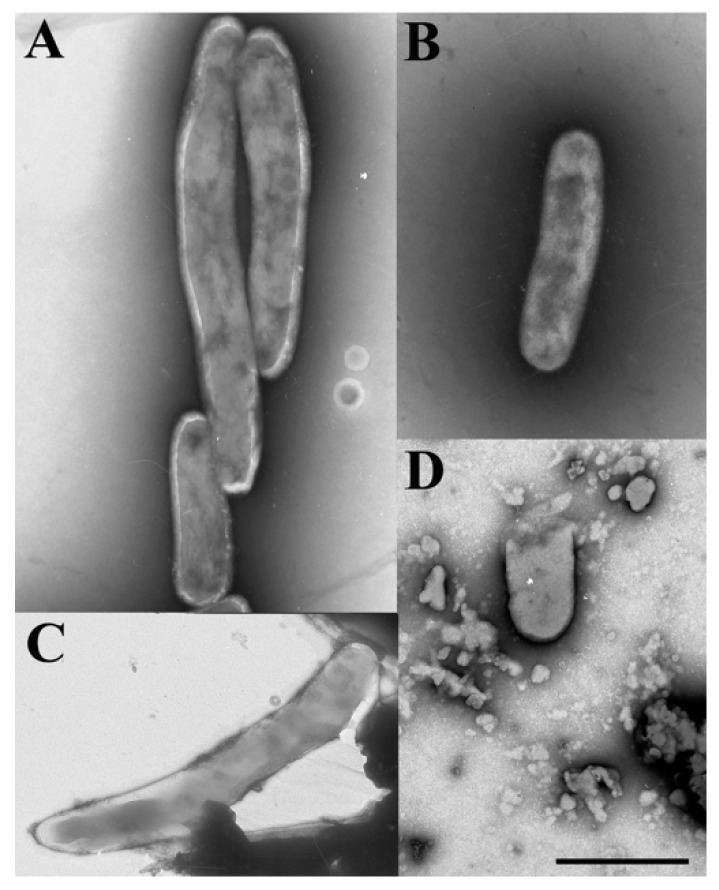
Transmission electron microscopy of *M. tuberculosis* H37Rv bacterial cells. Control bacterial cells were grown for 4 days in enriched Dubois medium (**A**) or in enriched Dubois medium containing DMSO/Twin-80/H_2_O solution without compound **3** (**B**). Bacterial cells after treatment with compound **3** (50 µg/mL) for 4 days (**C** and **D**). Scale bar = 1 µm.

Figure 4 shows electron micrographs of control *M. tuberculosis* H37Rv cells grown for 4 days in enriched Dubois medium (A) and in the presence of DMSO/Twin-80/H_2_O buffer without compound **3** (B). The morphology of bacterial cells grown in the medium containing compound **3** was markedly different. As shown in Figure 4C,D, the bacterial wall was damaged (Figure 4C) or completely destroyed (Figure 4D). This observation suggests that inactivation of *M. tuberculosis* may occur due to the interaction of compound **3** with the bacterial wall and its subsequent destruction. The main difference of *Mycobacteria* from other bacteria is their thick cell wall containing unique lipids—mycolic acids [54,55]. Thus speculatation that the synthesized compounds selectively act on mycolic acids of the *Mycobacteria* cell wall or, like isoniazid, block the pathways involved in mycolic acids biosynthesis [56] was logical. More testing however needs to be performed to confirm this observation, but if this phenomenon is subsequently confirmed for the majority of species of gram-positive and gram-negative bacteria, then these compounds may prove useful due to the lack of effect on the natural microflora of the gut bacteria.

## 3. Materials and Methods

### 3.1. Chemistry

The reactions were performed with the use of commercial reagents acquired from Acros (Geel, Belgium), Aldrich (St. Louis, MO, USA) and Fluka (Bucharest, Romania); anhydrous solvents were purified according to the standard procedures. Column chromatography was performed on Silica Gel 60 0.040–0.063 mm (Merck, Darmstadt, Germany) columns. Thin layer chromatography (TLC) was performed on Silica Gel 60 F_254_ aluminum-backed plates (Merck). Preparative layer chromatography (PLC) was performed on Silica Gel 60 F_254_ glass-backed plates (Merck). NMR spectra were registered on an AMX III-400 spectrometer (Bruker, Newark, Germany) with the working frequency of 400 MHz for ^1^H-NMR (Me_4_Si as an internal standard for organic solvents) and 100.6 MHz for ^13^C-NMR (with carbon-proton interaction decoupling). High resolution mass spectra (HRMS) were registered on a Bruker Daltonics micrOTOF or a Bruker micrOTOF II instrument using electrospray ionization (ESI HRMS) (Bruker Daltonics, Hamburg, Germany). The measurements were done in positive ion mode (interface capillary voltage 4500 V) in a mass range from *m*/*z* 50 to *m*/*z* 3000 Da; external or internal calibration was done with ESI Tuning Mix™ (Agilent Technologies, Santa Clara, CA, USA). Nitrogen was applied as a dry gas (6 L/min); the interface temperature was set at 180 °C, nebulizer pressure: 0.4 Bar; flow rate: 3 µL/min. Samples were injected into the mass spectrometer chamber from the Agilent 1260 HPLC system equipped with an Agilent Poroshell 120 EC-C18 column (3.0 × 50 mm; 2.7 µm) and an identically packed security guard, using an autosampler. The samples were injected from acetonitrile (LC-MS grade) solution. The column temperature was 30 °C and 5 μL of the sample solution was injected. The column was eluted in a gradient of concentrations of A (acetonitrile) in B (water) with the flow rate of 400 µL/min in the following gradient parameters: 0–15% A for 6.0 min, 15%–85% A for 1.5 min, 85%–0% A for 0.1 min, 0% A for 2.4 min.

*(±)-1-(4′-Hydroxy-2′-cyclopenten-1′-yl)-thymine* (**13**). Epoxycyclopentene (0.8 g, 10 mmol) in freshly distilled THF (15 mL) and tetrakis(triphenylphosphine)palladium (0) [Pd(PPh_3_)_4_] (308 mg; 0.26 mmol) were added to a stirred solution of thymine (1 g, 8.0 mmol) in anhydrous DMF (40 mL). The reaction mixture was stirred at room temperature for 18 h, then concentrated under vacuum, and the residue dissolved in 98:2 CHCl_3_:MeOH (5 mL) and loaded onto a silica gel column. Elution with a 98:2 to 95:5 CHCl_3_:MeOH gave compound (±)-**13** as a white powder (600 mg, 36%): R*f* 0.32 (95:5 CHCl_3_:MeOH). ^1^H-NMR (DMSO-*d*_6_): 11.20 (1H, br. s, NH), 7.28 (1H, s, H-6), 6.14–6.11 (1H, m, H-2′), 5.79–5.77 (1H, m, H-3′), 5.39–5.36 (1H, m, H-1′), 5.20 (1H, d, *J* = 6.0 Hz, OH), 4.63–4.61 (1H, m, H-4′), 2.74–2.70 (1H, m, H-а5′), 1.76 (3H, s, 5-CH_3_), 1.37–1.33 (1H, m, H-b5′). ^13^C-NMR (DMSO-*d*_6_): 164.3, 151.3 (C-4, C-2), 140.4 (C-6), 137.7 (C-3′), 131.4 (C-2′), 109.9 (C-5), 73.8 (C-4′), 58.3 (C-1′), 39.9 (C-5′), 12.6 (CH_3_).

*(±)-1-(4′-Hydroxycyclopent-1′-yl)-thymine* (**14**). To a solution of (±)-**13** (0.6 g, 3 mmol) in anhydrous MeOH (15 mL) 0.1 g 10% Pd/C was added under H_2_ atmosphere and the reaction mixture was stirred at room temperature under H_2_ atmosphere for 18 h. The mixture was filtrated through a pad of Celite and the filtrate was evaporated to dryness. The residue was dissolved in 98:2 CHCl_3_:MeOH (5 mL) and loaded onto a silica gel column eluting with the same solvent system, followed by 95:5 CHCl_3_:MeOH to give compound (±)-**14** as an off white powder (510 mg, 81%): R*f* 0.35 (95:5 CHCl_3_:MeOH). ^1^H-NMR (CDCl_3_): 8.77 (1H, br. s, NH), 7.48 (1H, s, H-6), 5.03–4.95 (1H, m, H-1′), 4.41 (1H, m, H-4′), 2.39 (1H, d, *J* = 8.0 Hz, OH), 2.36–2.30 (1H, m, H-а5′), 2.20–2.13, 2.00–1.98 and 1.73–1.71 (4H, 3m, H-2′and H-3′), 1.93 (3H, s, 5-CH_3_), 1.70–1.67 (1H, m, H-b5′). ^13^C-NMR (CDCl_3_): 164.2, 151.2 (C-4, C-2), 139.9 (C-6), 109.9 (C-5), 73.6 (C-4′), 58.3 (C-1′), 39.9 (C-5′), 31.8 and 29.9(C-2′ and C-3′), 12.6 (CH_3_).

*(±)-1-(4′-Acetoxycyclopent-1′-yl)-thymine* (**15**). To a solution of (±)-**14** (0.5 g, 2.4 mmol) in anhydrous pyridine (15 mL), Ac_2_O (0.275 mL, 2.9 mmol) was added. The reaction mixture was stirred at room temperature for 18 h, concentrated under vacuum, and the residue dissolved in CHCl_3_ (5 mL), loaded onto a silica gel column. Elution with CHCl_3_ followed by 98:2 CHCl_3_:MeOH provided compound (±)-**15** as a white powder (0.5 g, 83%): R*f* 0.36 (elution with 98:2 CHCl_3_:MeOH). ^1^H-NMR (CDCl_3_): 9.13 (1H, br. s, NH), 7.21 (1H, s, H-6), 5.23–5.20 (1H, m, H-1′), 5.14–5.10 (1H, m, H-4′), 2.54–2.47 (1H, m, H-а5′), 2.20–2.16, 2.00–1.98 and 1.87–1.82 (4H, 3m, H-2′and H-3′), 2.08 (3H, s, Ac), 1.96 (3H, s, 5-CH_3_), 1.78–1.73 (1H, m, H-b5′). ^13^C-NMR (CDCl_3_): 170.0 (C(O) acetyl), 163.7, 151.2 (C-4, C-2), 136.7 (C-6), 111.4 (C-5), 74.8 (C-4’), 53.9 (C-1′), 37.8 (C-5′), 31.8 and 29.9, (C-2′ and C-3′), 21.3 and 12.8 (2CH_3_).

*(±)-1-(4′-acetoxycyclopent-1′-yl)-5-(bromomethyl)uracil* (**16**). A solution of Br_2_ (0.2 mL, 2.4 mmol) in DCE (5 mL) was added dropwise over a 3 h period with stirring to a boiling solution of (±)-**15** (0.25 g, 1 mmol) in DCE (10 mL) with concomitant irradiation using an incandescent 300 W lamp under an argon atmosphere. The progress of the reaction was monitored by TLC in EtOAc/hexane (1:1). The mixture was evaporated under vacuum, coevaporated with toluene (3 × 10 mL), the crude **16** dissolved in DMF (5 mL) and used without further purification in subsequent reactions.

#### 3.1.1. General Procedure for the Synthesis of (±)-1-(4′-Acetoxycyclopent-1′-yl)-5-alkyloxy-methyluracils **1**–**3**

The corresponding alcohol (1.5 mmol) was added to a solution of 1-(4′-acetoxycyclopent-1′-yl)-5-(bromomethyl)uracil (**13**, 0.5 mmol) in dry DMF (10 mL). The mixture was stirred for 48 h at 37 °C under argon atmosphere, evaporated under vacuum, and the residue dissolved in CHCl_3_ (5 mL), loaded onto a silica gel column, and eluted with CHCl_3_:MeOH (98:2) to provide the crude products which were purified as described below.

*(±)-1-(4′-Acetoxycyclopent-1′-yl)-5-decyloxymethyluracil* (**1**). Crude **1** was purified by PLC eluting with EtOAc/hexane (2:1) to give compound (±)-**1** as a white powder (92 mg, 45%): R*f* 0.40 (98:2 CHCl_3_:MeOH). ^1^H-NMR (CDCl_3_): 8.76 (1H, br. s, NH), 7.49 (1H, s, H-6), 5.23–5.21 (1H, m, H-1′), 5.15–5.11 (1H, m, H-4′), 4.26 (2H, s, 5-CH_2_O), 3.51 (2H, t, *J* = 8.0 Hz, CH_2_CH_2_(CH_2_)_7_CH_3_), 2.55–2.47 (1H, m, H-а5′), 2.22–2.18, 2.01–1.99 and 1.87–1.83 (4H, 3m, H-2′ and H-3′), 2.08 (3H, s, Ac), 1.78–1.72 (1H, m, H-b5′), 1.61–1.56 (2H, m, CH_2_CH_2_(CH_2_)_7_CH_3_), 1.29–1.25 (14H, m, CH_2_CH_2_(CH_2_)_7_CH_3_), 0.86 (3H, t, *J* = 8 Hz, CH_2_CH_2_(CH_2_)_7_CH_3_). ^13^C-NMR (CDCl_3_): 170.3 (C(O) acetyl), 162.2, 150.9 (C-4, C-2), 138.0 (C-6), 112.9 (C-5), 74.8 (C-4′), 71.6 (5-CH_2_O), 65.0 (CH_2_CH_2_(CH_2_)_7_CH_3_), 54.4 (C-1′), 38.0 (C-5′), 32.0, 31.9, 30.0, 29.8, 29.6 × 2, 29.5, 29.4, 26.2, 22.7, 21.3 (C-2′, C-3′, (CH_2_)_8,_ CH_3_C(O)), 14.2 (CH_3_). HRMS: found **m/*z* 409.2698, calculated C_22_H_36_N_2_O_5_ [M + H]^+^ 409.26697.

*(±)-1-(4′-Acetoxycyclopent-1′-yl)-5-undecyloxymethyluracil* (**2**). Crude **2** was purified by PLC eluting with EtOAc/hexane (2:1) mixture to give compound (±)-**2** as a white powder (99 mg, 47%): R*f* 0.41 (98:2 CHCl_3_:MeOH). ^1^H-NMR (CDCl_3_): 8.58 (1H, br. s, NH), 7.49 (1H, s, H-6), 5.25–5.20 (1H, m, H-1′), 5.17–5.11 (1H, m, H-4′), 4.26 (2H, s, 5-CH_2_O), 3.52 (2H, t, *J* = 8.0 Hz, CH_2_CH_2_(CH_2_)_8_CH_3_), 2.58–2.47 (1H, m, H-а5′), 2.22–2.18, 2.03–1.99 and 1.88–1.83 (4H, 3m, H-2′and H-3′), 2.09 (3H, s, Ac), 1.79–1.72 (1H, m, H-b5′), 1.62–1.58 (2H, m, CH_2_CH_2_(CH_2_)_8_CH_3_), 1.30–1.26 (16H, m, CH_2_CH_2_(CH_2_)_8_CH_3_), 0.87 (3H, t, *J* = 8.0 Hz, CH_2_CH_2_(CH_2_)_8_CH_3_). ^13^C-NMR (CDCl_3_): 170.2 (C(O) acetyl), 162.1, 150.9 (C-4, C-2), 138.0 (C-6), 112.8 (C-5), 74.7 (C-4’), 71.5 (5-CH_2_O), 64.9 (CH_2_CH_2_(CH_2_)_8_CH_3_), 54.3 (C-1′), 37.9 (C-5′), 31.9, 31.8, 29.9, 29.6 × 3, 29.5, 29.3, 29.0, 26.1, 22.7, 21.2 (C-2′, C-3′, (CH_2_)_9,_ CH_3_C(O)), 14.1 (CH_3_). HRMS: found **m/*z* 423.2853, calculated C_23_H_38_N_2_O_5_ [M + H]^+^ 423.2853.

*(±)-1-(4′-Acetoxycyclopent-1′-yl)-5-dodecyloxymethyluracil* (**3**). Crude **3** was purified by PLC eluting with EtOAc/hexane (2:1) to give compound (±)-**3** as a white powder (100 mg, 46%): R*f* 0.42 (elution with 98:2 CHCl_3_:MeOH). ^1^H-NMR (CDCl_3_): 8.92 (1H, br. s, NH), 7.43 (1H, s, H-6), 5.18-5.14 (1H, m, H-1′), 5.13-5.06 (1H, m, H-4′), 4.20 (2H, s, 5-CH_2_O), 3.46 (2H, t, J=8 Hz, CH_2_CH_2_(CH_2_)_9_CH_3_), 2.50–2.42 (1H, m, H-а5′), 2.16–2.12, 1.96–1.94 and 1.81–1.77 (4H, 3m, H-2′and H-3′), 2.03 (3H, s, Ac), 1.72–1.67 (1H, m, H-b5′), 1.57–1.50 (2H, m, CH_2_CH_2_(CH_2_)_9_CH_3_), 1.25–1.19 (18H, m, CH_2_CH_2_(CH_2_)_9_CH_3_), 0.86 (3H, t, *J* = 8.0 Hz, CH_2_CH_2_(CH_2_)_9_CH_3_). ^13^C-NMR (CDCl_3_): 170.3 (C(O) acetyl), 162.4, 151.1 (C-4, C-2), 138.1 (C-6), 113.0 (C-5), 74.9 (C-4′), 71.7 (5-CH_2_O), 65.0 (CH_2_CH_2_(CH_2_)_8_CH_3_), 54.5 (C-1′), 38.1 (C-5′), 32.1, 32.0, 30.1, 29.8 × 5, 29.6, 29.5, 26.3, 22.8, 21.4 (C-2′, C-3′, (CH_2_)_10,_ CH_3_C(O)), 14.2 (CH_3_). HRMS: found **m/*z* 437.3004, calculated C_24_H_40_N_2_O_5_ [M + NH]^+^ 437.3010.

*(±)-1-(4′-Acetoxycyclopent-1′-yl)-5-(azidomethyl)uracil* (**17**). Sodium azide (160 mg, 2.5 mmol) was added to a solution of 1-(4′-acetoxycyclopent-1′-yl)-5-(bromomethyl)uracil (**13**) (0.5 mmol) in dry DMF (10 mL). The mixture was stirred for 48 h at 37 °C under argon atmosphere, evaporated under vacuum, and the residue dissolved in CHCl_3_ (5 mL), applied onto a silica gel column, and eluted with CHCl_3_:MeOH (98:2) to give crude product **17**. It was then purified further by PLC eluted with EtOAc/hexane (1:1) to provide the desired compound **17** as off white solid (143 mg, 97.5%). ^1^H-NMR (CDCl_3_): 8.43 (1H, br. s, NH), 7.54 (1H, s, H-6), 5.31–5.27 (1H, m, H-1′), 5.22–5.17 (1H, m, H-4′), 4.23 (2H, s, 5-CH_2_), 2.60–2.53 (1H, m, H-а5′), 2.31–2.27, 2.10–2.05 and 1.93–1.85 (4H, 3m, H-2′and H-3′), 2.07 (3H, s, Ac), 1.81–1.75 (1H, m, H-b5′).

#### 3.1.2. General Procedure for the Synthesis of (±)-1-(4′-Acetoxycyclopent-1′-yl)-5-[(4-alkyl)-1,2,3-triazol-1-yl]methyluracils **7**–**9**

To a solution of azide **17** (143 mg, 0.49 mmol) and corresponding 1-alkyne (0.75 mmol) in dichloromethane (2 mL), copper sulphate (12.4 mg, 0.05 mmol), sodium ascorbate (30 mg, 0.15 mmol) and H_2_O (2 mL) were added. The reaction mixture was stirred for 17 h at room temperature. The solvents were evaporated under vacuum and the crude products were purified by PLC eluting with EtOAc/hexane (2:1) to give compounds **7**–**9**.

*(±)-1-(4′-Acetoxycyclopent-1′-yl)-5-[(4-octyl)-1,2,3-triazol-1-yl]methyluracil* (**7**). White powder (100 mg, 47.5%). R*f* 0.28 (EtOAc/hexane (2:1)).^1^H-NMR (CDCl_3_): 9.53 (1H, s, NH), 7.82 (1H, s, H-6), 7.60 (1H, s, H^triazol^), 5.03–5.20 (3H, m, H-1′and 5-CH_2_N), 5.16–5.08 (1H, m, H-4′), 2.71 (2H, t, *J* = 8.0 Hz, CH_2_CH_2_(CH_2_)_5_CH_3_), 2.59–2.49 (1H, m, H-а5′), 2.20–2.17, 2.03–1.99 and 1.90–1.79 (4H, 3m, H-2′and H-3′), 2.18 (3H, s, Ac), 1.76–1.65 (1H, m, H-b5′), 1.65–1.59 (2H, m, CH_2_CH_2_(CH_2_)_5_CH_3_), 1.29–1.24 (10H, m, CH_2_CH_2_(CH_2_)_5_CH_3_), 0.88 (3H, t, J=8 Hz, CH_2_CH_2_(CH_2_)_5_CH_3_). ^13^C-NMR (CDCl_3_): 170.4 (C(O) acetyl), 162.4, 150.5 (C-4, C-2), 148.7 (C-5^triazole^), 142.4 (C-6), 122.4 (C-4^triazole^), 109.0 (C-5), 74.4 (C-4′), 55.2 (C-1′), 46.6 (5-CH_2_N), 38.1 (C-5’), 31.9, 31.8, 30.2, 29.8, 29.3 × 3, 25.4, 22.7, 21.5 (C-2′, C-3′, (CH_2_)_7,_ CH_3_C(O)), 14.1 (CH_3_). HRMS: found **m/*z* 432.2601, calculated C_22_H_33_N_5_O_4_ [M + H]^+^ 432.2605.

*(±)-1-(4′-Acetoxycyclopent-1′-yl)-5-[(4-decyl)-1,2,3-triazol-1-yl]methyluracil* (**8**), white powder (143 mg, 63.5%). R*f* 0.30 (EtOAc/hexane (2:1)).^1^H-NMR (CDCl_3_): 9.03 (1H, s, NH), 7.78 (1H, s, H-6), 7.55 (1H, s, H^triazol^), 5.26-5.21 (3H, m, 5-CH_2_N and H-1′), 5.09–5.05 (1H, m, H-4′), 2.68 (2H, t, *J* = 8.0 Hz, CH_2_CH_2_(CH_2_)_7_CH_3_), 2.56–2.48 (1H, m, H-а5′), 2.22–2.17, 2.01–1.99 and 1.89–1.80 (4H, 3m, H-2′and H-3′), 2.15 (3H, s, Ac), 1.76–1.69 (1H, m, H-b5′), 1.66–1.62 (2H, m, CH_2_CH_2_(CH_2_)_7_CH_3_), 1.30–1.24 (14H, m, CH_2_CH_2_(CH_2_)_7_CH_3_), 0.86 (3H, t, *J* = 8.0 Hz, CH_2_CH_2_(CH_2_)_7_CH_3_). ^13^C-NMR (CDCl_3_): 170.4 (C(O) acetyl), 162.5, 150.7 (C-4, C-2), 148.7 (C-5^triazole^), 142.2 (C-6), 122.1 (C-4^triazole^), 109.5 (C-5), 74.5 (C-4′), 55.2 (C-1′), 45.2 (5-CH_2_N), 38.2 (C-5′), 32.0, 31.9, 30.3, 29.7 × 2, 29.4 × 4, 25.7, 22.8, 21.5 (C-2′, C-3′, (CH_2_)_9,_ CH_3_C(O)), 14.2 (CH_3_). HRMS: found **m/*z* 460.2919, calculated C_24_H_37_N_5_O_4_ [M + H]^+^ 460.2918.

*(**±**)-1-(4′-Acetoxycyclopent-1′-yl)-5-[(4-dodecyl)-1,2,3-triazol-1-yl]methyluracil* (**9**), white powder (127 mg, 53%). R*f* 0.32 (EtOAc/hexane (2:1)).^1^H-NMR (CDCl_3_): 9.30 (1H, s, NH), 7.78 (1H, s, H-6), 7.55 (1H, s, H^triazol^), 5.21 (3H, m, 5-CH_2_N and H-1′), 5.09–5.05 (1H, m, H-4′), 2.67 (2H, t, *J* = 8.0 Hz, CH_2_CH_2_(CH_2_)_9_CH_3_), 2.55–2.47 (1H, m, H-а5′), 2.20–2.17, 2.02–1.99 and 1.93–1.83 (4H, 3m, H-2′ and H-3′), 2.15 (3H, s, Ac), 1.76–1.70 (1H, m, H-b5′), 1.65–1.61 (2H, m, CH_2_CH_2_(CH_2_)_9_CH_3_), 1.25–1.22 (18H, m, CH_2_CH_2_(CH_2_)_9_CH_3_), 0.86 (3H, t, *J* = 8.0 Hz, CH_2_CH_2_(CH_2_)_9_CH_3_). ^13^C-NMR (CDCl_3_): 170.4 (C(O) acetyl), 162.5, 150.7 (C-4, C-2), 148.7 (C-5^triazole^), 142.1 (C-6), 122.0 (C-4^triazole^), 109.5 (C-5), 74.4 (C-4′), 55.1 (C-1′), 46.1 (5-CH_2_N), 38.1 (C-5′), 32.0, 31.8, 30.2, 29.7 × 3, 29.6, 29.4 × 4, 25.6, 22.7, 21.4 (C-2′, C-3′, (CH_2_)_11,_ CH_3_C(O)), 14.2 (CH_3_). HRMS: found **m/*z* 488.2232, calculated C_26_H_41_N_5_O_4_ [M + H]^+^ 488.2232.

#### 3.1.3. General Procedure for the Synthesis of (±)-1-(4′-Hydroxycyclopent-1′-yl)-5-alkyloxy-methyluracils **4**–**6** and (±)-1-(4′-hydroxycyclopent-1′-yl)-5-[(4-alkyl)-1,2,3-triazol-1-yl]methyl-uracils **10**–**12**

To a stirred solution of the corresponding acetylated compound **1**–**3**, **7**–**9** (0.15 mmol) in MeOH (5 ml) K_2_CO_3_ (52 mg; 0.38 mmol) was added and refluxed during 2–4 h. The progress of the reaction was monitored by TLC in 95:5 CHCl_3_:MeOH. The reaction mixture was concentrated in vacuum and the residue purified by PLC eluting with 9:1 CHCl_3_-MeOH to give the desired compounds.

*(±)-1-(4′-Hydroxycyclopent-1′-yl)-5-decyloxymethyluracil* (**4**), white solid (47 mg, 85 %): R*f* 0.47 (9:1 CHCl_3_:MeOH). ^1^H-NMR (CDCl_3_): 8.72 (1H, br. s, NH), 7.73 (1H, s, H-6), 5.00–4.92 (1H, m, H-1′), 4.39 (1H, m, H-4′), 4.24 (2H, s, 5-CH_2_O), 3.50 (2H, t, J = 8 Hz, CH_2_CH_2_(CH_2_)_7_CH_3_), 2.36–2.34 (1H, m, H-а5′), 2.19–2.16, 1.99–1.89 (4H, 2m, H-2′and H-3′), 1.77–1.72 (1H, m, H-b5′), 1.60–1.57 (2H, m, CH_2_CH_2_(CH_2_)_7_CH_3_), 1.28–1.25 (14H, m, CH_2_CH_2_(CH_2_)_7_CH_3_), 0.86 (3H, t, *J* = 8.0 Hz, CH_2_CH_2_(CH_2_)_7_CH_3_). ^13^C-NMR (CDCl_3_): 162.5, 151.0 (C-4, C-2), 140.8 (C-6), 112.6 (C-5), 72.2 (C-4′), 71.5 (5-CH_2_O), 65.0 (CH_2_CH_2_(CH_2_)_7_CH_3_), 56.4 (C-1′), 40.5 (C-5′), 35.3, 32.0, 29.8, 29.7 × 3, 29.6, 29.4, 26.2, 22.8 (C-2′, C-3′, (CH_2_)_8_), 14.2 (CH_3_). HRMS: found **m/*z* 367.2592, calculated C_20_H_34_N_2_O_4_ [M + H]^+^ 367.2591.

*(±)-1-(4′-Hydroxycyclopent-1′-yl)-5-undecyloxymethyluracil* (**5**), white solid (51 mg, 88 %): R*f* 0.49 (9:1 CHCl_3_:MeOH). ^1^H-NMR (CDCl_3_): 8.56 (1H, br. s, NH), 7.72 (1H, s, H-6), 4.98-4.94 (1H, m, H-1′), 4.40 (1H, m, H-4′), 4.24 (2H, s, 5-CH_2_O), 3.50 (2H, t, J = 8 Hz, CH_2_CH_2_(CH_2_)_8_CH_3_), 2.41–2.33 (2H, m, H-а5′, OH), 2.21–2.14, 2.05–1.96, 1.94–1.87 and 1.78–1.75 (4H, 4m, H-2′and H-3′), 1.74–1.69 (1H, m, H-b5′), 1.62-1.55 (2H, m, CH_2_CH_2_(CH_2_)_8_CH_3_), 1.29–1.25 (16H, m, CH_2_CH_2_(CH_2_)_8_CH_3_), 0.87 (3H, t, *J* = 8.0 Hz, CH_2_CH_2_(CH_2_)_8_CH_3_). ^13^C-NMR (CDCl_3_): 162.4, 151.0 (C-4, C-2), 140.7 (C-6), 112.6 (C-5), 72.2 (C-4′), 71.5 (5-CH_2_O), 65.0 (CH_2_CH_2_(CH_2_)_8_CH_3_), 56.4 (C-1′), 40.5 (C-5′), 35.3, 32.00, 29.8, 29.7 × 4, 29.6, 29.4, 26.2, 22.8 (C-2′, C-3′, (CH_2_)_9_), 14.2 (CH_3_). HRMS: found **m/*z* 381.2749, calculated C_21_H_36_N_2_O_4_ [M + H]^+^ 381.2748.

*(±)-1-(4′-Hydroxycyclopent-1′-yl)-5-dodecyloxymethyluracil* (**6**), white solid (51.5 mg, 87 %): R*f* 0.50 (9:1 CHCl_3_:MeOH). ^1^H-NMR (CDCl_3_): 8.60 (1H, br. s, NH), 7.72 (1H, s, H-6), 4.97–4.94 (1H, m, H-1′), 4.40 (1H, m, H-4′), 4.24 (2H, s, 5-CH_2_O), 3.50 (2H, t, J = 8 Hz, CH_2_CH_2_(CH_2_)_9_CH_3_), 2.41–2.33 (1H, m, H-а5′), 2.21–2.14, 2.05–1.97, 1.94–1.87 and 1.75 (4H, 4m, H-2′and H-3′), 1.75–1.67 (1H, m, H-b5′), 1.62–1.55 (2H, m, CH_2_CH_2_(CH_2_)_9_CH_3_), 1.29–1.25 (18H, m, CH_2_CH_2_(CH_2_)_9_CH_3_), 0.86 (3H, t, *J* = 8.0 Hz, CH_2_CH_2_(CH_2_)_9_CH_3_). ^13^C-NMR (CDCl_3_): 162.4, 151.0 (C-4, C-2), 140.7 (C-6), 112.6 (C-5), 72.2 (C-4′), 71.5 (5-CH_2_O), 65.0 (CH_2_CH_2_(CH_2_)_8_CH_3_), 56.5 (C-1′), 40.4 (C-5′), 35.3, 32.0, 29.7 × 5, 29.6, 29.5, 29.4, 26.2, 22.8 (C-2′, C-3′, (CH_2_)_10_), 14.2 (CH_3_). HRMS: found **m/*z* 395.2904, calculated C_22_H_38_N_2_O_4_ [M + H]^+^ 395.2904.

*(±)-1-(4′-Hydroxycyclopent-1′-yl)-5-[(4-octyl)-1,2,3-triazol-1-yl]methyluracil* (**10**), white solid (54 mg, 92%): R*f* 0.49 (9:1 CHCl_3_:MeOH). ^1^H-NMR (CDCl_3_): 9.41 (1H, s, NH), 8.22 (1H, s, H-6), 7.60 (1H, s, H^triazole^), 5.28–5.19 (2H, m, 5-CH_2_N), 5.13–5.07 (1H, m, H-1′), 4.40 (1H, m, H-4′), 2.67 (2H, t, *J* = 8.0 Hz, CH_2_CH_2_(CH_2_)_5_CH_3_), 2.33–2.25 (1H, m, H-а5′), 2.23–2.19, 1.98–1.84 and 1.67–1.64 (4H, 3m, H-2′and H-3′), 1.74–1.70 (1H, m, H-b5′), 1.62–1.57 (2H, m, CH_2_CH_2_(CH_2_)_5_CH_3_), 1.28–1.23 (10H, m, CH_2_CH_2_(CH_2_)_5_CH_3_), 0.85 (3H, t, *J* = 8.0 Hz, CH_2_CH_2_(CH_2_)_5_CH_3_). ^13^C-NMR (CDCl_3_): 162.8, 151.0 (C-4, C-2), 148.3 (C-5^triazole^), 144.2 (C-6), 122.5 (C-4^triazole^), 109.1 (C-5), 72.0 (C-4′), 55.7 (C-1′), 46.5 (5-CH_2_N), 41.0 (C-5′), 35.2, 31.9, 30.7, 29.8, 29.4 × 2, 29.3, 25.5, 22.7 (C-2′, C-3′, (CH_2_)_7_), 14.1 (CH_3_). HRMS: found **m/*z* 390.2501, calculated C_20_H_31_N_5_O_3_ [M + H]^+^ 390.2500.

*(±)-1-(4′-Hydroxycyclopent-1′-yl)-5-[(4-decyl)-1,2,3-triazol-1-yl]methyluracil* (**11**), white solid (53 mg, 84%): R*f* 0.50 (9:1 CHCl_3_:MeOH). ^1^H-NMR (CDCl_3_): 9.71 (1H, s, NH), 8.17 (1H, s, H-6), 7.54 (1H, s, H^triazole^), 5.24-5.19 (2H, m, 5-CH_2_N), 5.16-5.06 (1H, m, H-1’), 4.41–4.40 (1H, m, H-4′), 2.63 (2H, t, *J* = 8.0 Hz, CH_2_CH_2_(CH_2_)_7_CH_3_), 2.34–2.26 (1H, m, H-а5′), 2.23–2.17, 1.91–1.84 and 1.74–1.65 (4H, 3m, H-2′and H-3′), 1.77–1.73 (1H, m, H-b5′), 1.64–1.57 (2H, m, CH_2_CH_2_(CH_2_)_7_CH_3_), 1.28–1.23 (14H, m, CH_2_CH_2_(CH_2_)_7_CH_3_), 0.85 (3H, t, *J* = 8.0 Hz, CH_2_CH_2_(CH_2_)_7_CH_3_). ^13^C-NMR (CDCl_3_): 162.8, 150.2 (C-4, C-2), 148.2 (C-5^triazole^), 144.2 (C-6), 122.6 (C-4^triazole^), 109.0 (C-5), 72.0 (C-4′), 55.8 (C-1′), 46.7 (5-CH_2_N), 41.0 (C-5′), 35.2, 32.0, 30.7, 29.7, 29.6, 29.4 × 4, 25.5, 22.8 (C-2′, C-3′, (CH_2_)_9_), 14.2 (CH_3_). HRMS: found **m/*z* 418.2078, calculated C_22_H_35_N_5_O_3_ [M + H]^+^ 418.2813.

*(±)-1-(4′-Hydroxycyclopent-1′-yl)-5-[(4-dodecyl)-1,2,3-triazol-1-yl]methyluracil* (**12**), white solid (519 mg, 88%): R*f* 0.52 (9:1 CHCl_3_:MeOH). ^1^H-NMR (CDCl_3_): 9.02 (1H, s, NH), 8.11 (1H, s, H-6), 7.52 (1H, s, H^triazole^), 5.24–5.20 (2H, m, 5-CH_2_N), 5.16–5.04 (1H, m, H-1′), 4.42–4.40 (1H, m, H-4′), 2.65 (2H, t, *J* = 8.0 Hz, CH_2_CH_2_(CH_2_)_9_CH_3_), 2.34–2.28 (1H, m, H-а5′), 2.25–2.18, 1.94-1.83 and 1.69–1.66 (4H, 3m, H-2′and H-3′), 1.73–1.70 (1H, m, H-b5′), 1.64–1.59 (2H, m, CH_2_CH_2_(CH_2_)_9_CH_3_), 1.28–1.24 (18H, m, CH_2_CH_2_(CH_2_)_9_CH_3_), 0.86 (3H, t, *J* = 8.0 Hz, CH_2_CH_2_(CH_2_)_9_CH_3_). ^13^C-NMR (CDCl_3_): 162.6, 150.9 (C-4, C-2), 148.8 (C-5^triazole^), 143.8 (C-6), 122.0 (C-4^triazole^), 109.5 (C-5), 72.1 (C-4′), 55.8 (C-1’), 46.1 (5-CH_2_N), 40.9 (C-5’), 35.3, 32.0, 30.6, 29.7 × 4, 29.5 × 2, 29.4 × 2, 25.8, 22.8 (C-2′, C-3′, (CH_2_)_11_), 14.2 (CH_3_). HRMS: found **m/*z* 446.3127, calculated C_24_H_39_N_5_O_3_ [M + H]^+^ 446.3127.

### 3.2. Antibacterial Experiments

#### 3.2.1. Bacterial Strains

The *M. tuberculosis* ATCC 25177 and *M. bovis* ATCC 35737 strains used in these studies were purchased from the American Type Culture Collection (ATCC, Manassas, VA, USA). The laboratory *M.tuberculosis* H37Rv strain and the MS-115 clinical strain resistant to five first line anti-TB drugs (rifampicin, isoniazid, streptomycin, ethambutol, and pyrazinamide) were acquired from the Russian Central Tuberculosis Research Institute Collection (Moscow, Russia). The bacteria were propagated as recommended by the supplier. *B. subtilis* ATCC 6633, *S. aureus* INA 00761 (MRSA), *M. smegmatis* mc^2^155, *M. smegmatis* VKPM Ac 1339, *L. mesenteroides* VKPM B-4177, *P. aeruginosa* ATCC 27853, and *E. coli* ATCC 25922 were purchased from the collection of Gause Institute of New Antibiotics (Moscow, Russia).

#### 3.2.2. U-937 Cells

The U-937 cells used in the cytotoxicity evaluation were obtained from the AIDS Research and Reference Program (Germantown, MD, USA). The cells were cultured as specified by the supplier in RPMI-1640 supplemented with 10% heat inactivated fetal bovine serum, 1% penicillin/streptomycin solution and 1% l-glutamine.

#### 3.2.3. Determination of Minimal Inhibitory Concentration (MIC) against *M. tuberculosis* ATCC 25177

The susceptibility of the *M. tuberculosis* ATCC 25177 to the test compounds was evaluated by determining the MIC of each compound using a micro-broth dilution analysis [57,58,59,60]. A standardized inoculum was prepared by direct suspension of bacteria from a Lowenstein-Jensen slant to Middlebrook 7H9 broth with ADC enrichment. The broth culture was incubated for 26 days at 37 °C. Following the incubation, the culture was vortexed with glass beads resulting in an OD_595_ of 0.18 and then further diluted 1:20 to yield 5 × 10^6^ CFU/mL. One hundred microliters (100 μL) was added in triplicate wells of a 96-well plate containing 100 μL of test compound serially diluted 2-fold in the appropriate broth. This dilution scheme yielded final concentrations of the bacteria estimated to be 2.5 × 10^6^ CFU/mL. The plates were incubated for seven days at 37 °C at which time 30 µL of reazurin solution was added to each well and the cultures were incubated for an additional 24 h at 37 °C. Following the incubation, the plates were visually scored +/- for color change from blue (no growth) to pink (growth). The MIC for each compound was determined as the lowest compound dilution that completely inhibited microbial growth.

#### 3.2.4. Determination of MIC against *M. bovis* ATCC 35737

The susceptibility of *M. bovis* ATCC 35737 to the test compounds was evaluated by determining the MIC of each compound using a micro-broth dilution [57]. A standardized inoculum was prepared by direct suspension of bacteria from a Lowenstein-Jensen slant to Middlebrook 7H9 broth with ADC (albumin, dextrose, catalase) enrichment and 40 mM sodium pyruvate. The broth culture was incubated for 23 days at 37 °C. Following the incubation, the culture was vortexed with glass beads resulting in an OD_595_ = 0.203 which was further diluted 1:10 to yield 3 × 10^7^ CFU/mL. One-hundred microliters (100 μL) was added in triplicate wells of a 96-well plate containing 100 μL of test compound serially diluted 2-fold in the appropriate broth. This dilution scheme yielded final concentrations of the bacteria estimated to be 1.5 × 10^7^ CFU/mL. The plates were incubated for five days at 37 °C at which time 30 µL of reazurin solution was added to each well. The cultures were incubated for an additional 4 days at 37 °C at which time the plates were visually scored +/- for color change from blue (no growth) to pink (growth). The MIC for each compound was determined as the lowest compound dilution that completely inhibited microbial growth.

#### 3.2.5. Antituberculosis Tests with Virulent Strains of *M. tuberculosis*

The laboratory strain of *M. tuberculosis* H37Rv susceptible to anti-TB drugs and MDR strain *M. tuberculosis* MS-115 were used. The MDR strain is resistant toward five first line anti-TB drugs: rifampicin, isoniazid, streptomycin, ethambutol and pyrazinamide. The mycobacteria were transformed into a suspension of single cells at the same growth phase and normalized by CFU [61] and studied using an automated BACTEC MGIT 960 system (BD, Westfield, MA, USA) [62]. Tested compounds were dissolved in DMSO at a concentration of 40 mg/mL (stock solutions). Water and Tween-80 were added to the stock solutions to provide DMSO/Tween-80/H_2_O (5:0.5:4.5, *v*/*v*/*v*). The concentration of mycobacterial cells was 10^6^ CFU/mL. Each sample, including the control samples lacking the tested compound, were evaluated in triplicate as previously reported [28,29]. The antibacterial activity was analyzed with the TB Exist software [53]. The method is based on comparison of the bacterial growth in the samples, containing the tested compounds, and the control samples diluted 100 times if compared with the initially inoculated culture. The culture is considered susceptible (i.e., the compound is active) if the optical density of the control sample is 400 GU (growth units) and that of the tested sample is less than 100 GU. In addition, using the absolute concentrations method, a delay in the bacterial growth of the tested sample was measured in comparison with the control samples, which reflected a negative effect of the tested compounds at the concentrations lower than MIC on the bacterial viability. The measurements were carried out automatically every hour and registered with the Epicenter software (BD, Westfield, MA, USA).

#### 3.2.6. Antibacterial Effects

The antibacterial effects for each of the compounds as well as rifampicin, isoniazid, ciprofloxacin and amikacin were determined by the micro-broth delution method [63] against *B. subtilis* ATCC 6633, *S. aureus* INA 00761 (MRSA), *M. smegmatis* mc^2^155, *M. smegmatis* VKPM Ac 1339, *L. mesenteroides* VKPM B-4177, *P. aeruginosa* ATCC 27853, and *E. coli* ATCC 25922. These strains were purchased from the collection of Gause Institute of New Antibiotics. Two-fold serial dilutions of the compounds in nutrient medium No. 2 Gause (composition (%): glucose-1, peptone-0.5, tryptone-0.3, NaCl-0.5, pH 7.2–7.4) were performed to include concentrations ranging from 300 μg/mL to 1 μg/mL, and the test strains were cultured at 10^6^ cells/mL. All cultures were incubated at 37 °C with the exception of *L. mesenteroides* VKPM B-4177, which was incubated at 28 °C. The presence of growth and growth intensity were determined after one day in all the strains with the exception of *M. smegmatis*, which was evaluated after two days of growth.

#### 3.2.7. Evaluation of U-937 Cytotoxicity

U-937 cells plated at 2.5 × 10^3^ cells/well in a volume of 100 µL were incubated with serially diluted test compounds in a volume of 100 µL for 6 days at 37 °C/5% CO_2_. Following the incubation, the cells were stained with the tetrazolium dye XTT to determine cellular viability and the plates were read at 460/650 nm on a spectrophotometer. Toxicity values were calculated using linear regression analysis.

#### 3.2.8. Electron Microscopy

For electron microscopy *M. tuberculosis* H37Rv cells (10^9^ CFU/mL) were grown in enriched Dubois medium with compound **3** dissolved in of DMSO/Twin-80/H_2_O solution as described above. The final concentration of compound **3** was 60 µg/mL. In control experiments the cells were grown in pure enriched Dubois medium or in the presence of DMSO/Twin-80/H_2_O solution without compound **3**. After 4 days the cells were collected by centrifugation, washed in distilled water and fixed in 2.5% glutaraldehyde. Aliquotes (80 mkL) of cell suspensions were centrifuged in the small centrifuge chambers onto freshly glow-discharged carbon-collodion electron microscopic grids at 2500 g for 20 min [64,65]. Specimens were briefly rinsed in distilled water, stained for 1 min in 1% aqueous uranyl acetate solution [66], air dried and studied on a JEM-100CX electron microscope (JEOL, Peabody, MA, USA) in Core Facility “UNIQEM Collection” of Federal Research Centre “Fundamentals of Biotechnology” of the Russian Academy of Sciences.

## 4. Conclusions

In summary, twelve new 5′q5 ml-norcarbocyclic derivatives of 5-alkoxymethyl or 5-alkyl-triazolylmethyl uracil were synthesized. The antibacterial activity of the compounds was tested against different classes of bacteria, including both Gram-positive and Gram-negative strains. It was determined that this set of uracil derivatives possess selectivity towards *Mycobacteria*. Analogues **3**, **7** and **8** exhibited activities against two virulent strains of *M. tuberculosis*: laboratory sensitive strain H37Rv and the MDR clinical strain MS-115, which is known to be clinically resistant to five top antituberculosis drugs. Notably, analogue **7** was 2.5 times more active against the MDR strain. However, all three of the active compounds proved to be quite toxic to the monocytic cell line U937, thus, additional work needs to be undertaken to attenuate this toxicity and improve the activity. TEM studies of *M. tuberculosis* H37Rv bacterial cells treated with **3** as a representative of this set demonstrated destruction of the bacterial cell wall. The results of these studies thus suggest that the mechanism of action for these compounds is most likely related to interactions with the bacterial cell wall. Ongoing studies currently underway will hopefully provide more insight on this interesting finding and will be reported elsewhere as they become available.

## Figures and Tables

**Table 1:** Preliminary evaluation of antimycobacterial activity of compounds **1**–**12**.

Compound	*M. tuberculosis* ATCC 25177 MIC_99_^a^ (μg/mL)	*M. bovis* ATCC 35737 MIC_99_^a^ (μg/mL)	*M. smegmatis* mc^2^155 MIC_99_^a^ (μg/mL)	*M. smegmatis* VKPM Ac 1339 MIC_99_^a^ (μg/mL)
(±) **1**	409	409	>100	>100
(±) **2**	423	423	>100	>100
(±) **3**	54	54	67	67
(±) **4**	183	366	>100	>100
(±) **5**	191	381	>100	>100
(±) **6**	198	>395	67	67
(±) **7**	54	54	>100	>100
(±) **8**	29	57	67	6.7
(±) **9**	61	488	>100	>100
(±) **10**	98	195	>100	>100
(±) **11**	53	105	67	6.7
(±) **12**	56	446	>100	>100
Rifampicin	<0.2	<0.2	>100	10
Streptomycin	0.4	0.4	-	-
Kanamycin	1.6	1.6	-	-
Ciprofloxacin	-	-	1	2
Amikacin	-	-	<3.8	<3.8
Isoniazid	-	-	64	1

^a^ The concentration of the drug at which the growth of the bacteria is inhibited by 99%.

**Table 2:** The antimycobacterial activity * of 5′-norcarbocyclic derivatives of 5-substituted uracil and cytotoxicity to U937 cells.

Compound	*M. tuberculosis* H37Rv MIC_99_ (μg/mL)	*M. tuberculosis* MS-115 MIC_99_ (μg/mL)	U-937 TC_50_ (μg/mL)
(±) **3**	20	20	20.6
(±) **7**	50	20	23.7
(±) **8**	50	50	15.1
Rifampicin	1	>50	>500
Isoniazid	0.1	>100	-
Levofloxacin	1.5	1.5	-
Nonoxynol-9	-	-	5.9

* For more details see the Table in Supplementary Material.

## Supplementary Materials

Copies of the NMR spectra are available online.
