# Supplementary material for: Novel 5′-Norcarbocyclic Pyrimidine Derivatives as Antibacterial Agents
Source: Molecules. 2018 Nov 23;23(12):3069. doi: 10.3390/molecules23123069 (PMC6321083; doi:10.3390/molecules23123069)
Supplement: Supplementary file 1 [file molecules-23-03069-s001.pdf]

Novel 5'-norcarbocyclic pyrimidine derivatives as antibacterial agents

Anastasia L. Khandazhinskaya,[a,#] Liudmila A. Alexandrova,[a,#] Elena S. Matyugina,[a] Pavel N. Solyev,[a] Olga V. Efremenkova,[b] Karen W. Buckheit,[c] Maggie Wilkinson,[c] Robert W. Buckheit Jr,[c] Larisa N. Chernousova,[d] Tatiana G. Smirnova,[d] Sofya N. Andreevskaya,[d] Sergey N. Kochetkov, Katherine L. Seley-Radtke[e]

[a] *Engelhardt Institute of Molecular Biology RAS, Vavilova 32, Moscow, 119334, Russia*
[b] *Gause Institute of New Antibiotics, RAMS, Moscow, B. Pirogovskaya 11, 119021, Russia*
[c] *ImQuest BioSciences, Frederick, Maryland, USA*
[d] *Central Tuberculosis Research Institute, Yauzskaya Alley 2, Moscow, 107564, Russia*
[e] *Department of Chemistry & Biochemistry, University of Maryland, Baltimore County, Baltimore, MD 21250, USA*
[#] *equal contribution*

Supplementary materials

Table of contents

^{1}H NMR for compound **1** p. 2
^{13}C NMR for compound **1** p. 3
^{1}H NMR for compound **2** p. 4
^{13}C NMR for compound **2** p. 5
^{1}H NMR for compound **3** p. 6
^{13}C NMR for compound **3** p. 7
^{1}H NMR for compound **4** p. 8
^{13}C NMR for compound **4** p. 9
^{1}H NMR for compound **5** p.10
^{13}C NMR for compound **5** p.11
^{1}H NMR for compound **6** p.12
^{13}C NMR for compound **6** p.13
^{1}H NMR for compound **7** p.14
^{13}C NMR for compound **7** p.15
^{1}H NMR for compound **8** p.16
^{13}C NMR for compound **8** p.17
^{1}H NMR for compound **9** p.18
^{13}C NMR for compound **9** p.19
^{1}H NMR for compound **10** p.20
^{13}C NMR for compound **10** p.21
^{1}H NMR for compound **11** p.22
^{13}C NMR for compound **11** p.23
^{1}H NMR for compound **12** p.24
^{13}C NMR for compound **12** p.25
Table. The antimycobacterial activity of 5'-norcarbocyclic derivatives of 5-substituted uracil p.26

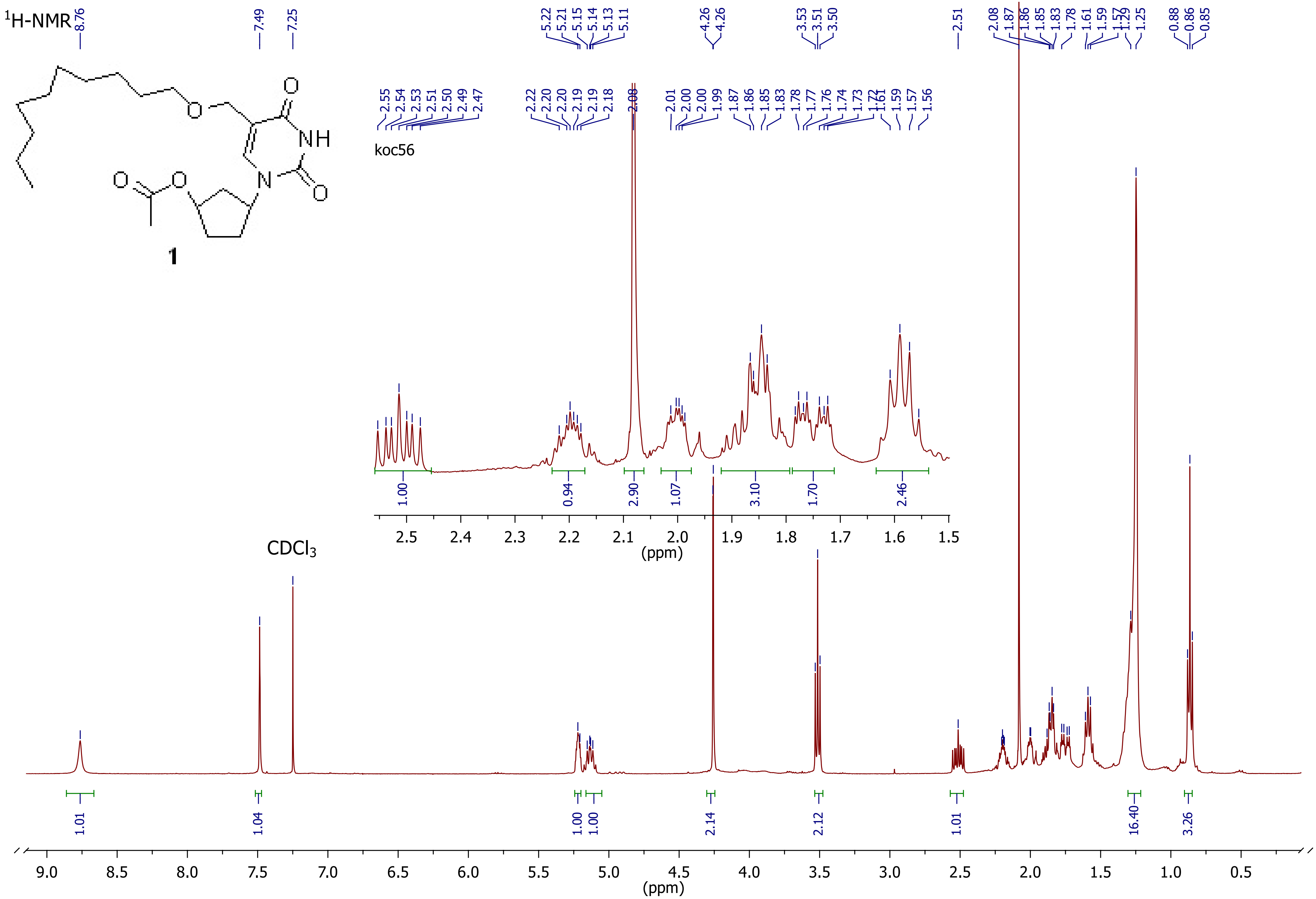

1H-NMR
1
koc56
CDCl3
8.76
7.49
7.25
5.22 5.21 5.15 5.14 5.13 5.11
4.26 4.26
3.53 3.51 3.50
2.51
2.08 1.87 1.86 1.85 1.83 1.78 1.61 1.59 1.57 1.29 1.25
0.88 0.86 0.85
2.55 2.54 2.53 2.51 2.50 2.49 2.47
2.22 2.20 2.20 2.19 2.19 2.18
2.08
2.01 2.00 2.00 1.99 1.87 1.86 1.85 1.83 1.78 1.77 1.76 1.74 1.73 1.72 1.61 1.59 1.57 1.56
1.00 0.94 2.90 1.07 3.10 1.70 2.46
2.5 2.4 2.3 2.2 2.1 2.0 1.9 1.8 1.7 1.6 1.5
(ppm)
1.01 1.04 1.00 1.00 2.14 2.12 1.01 16.40 3.26
9.0 8.5 8.0 7.5 7.0 6.5 6.0 5.5 5.0 4.5 4.0 3.5 3.0 2.5 2.0 1.5 1.0 0.5
(ppm)

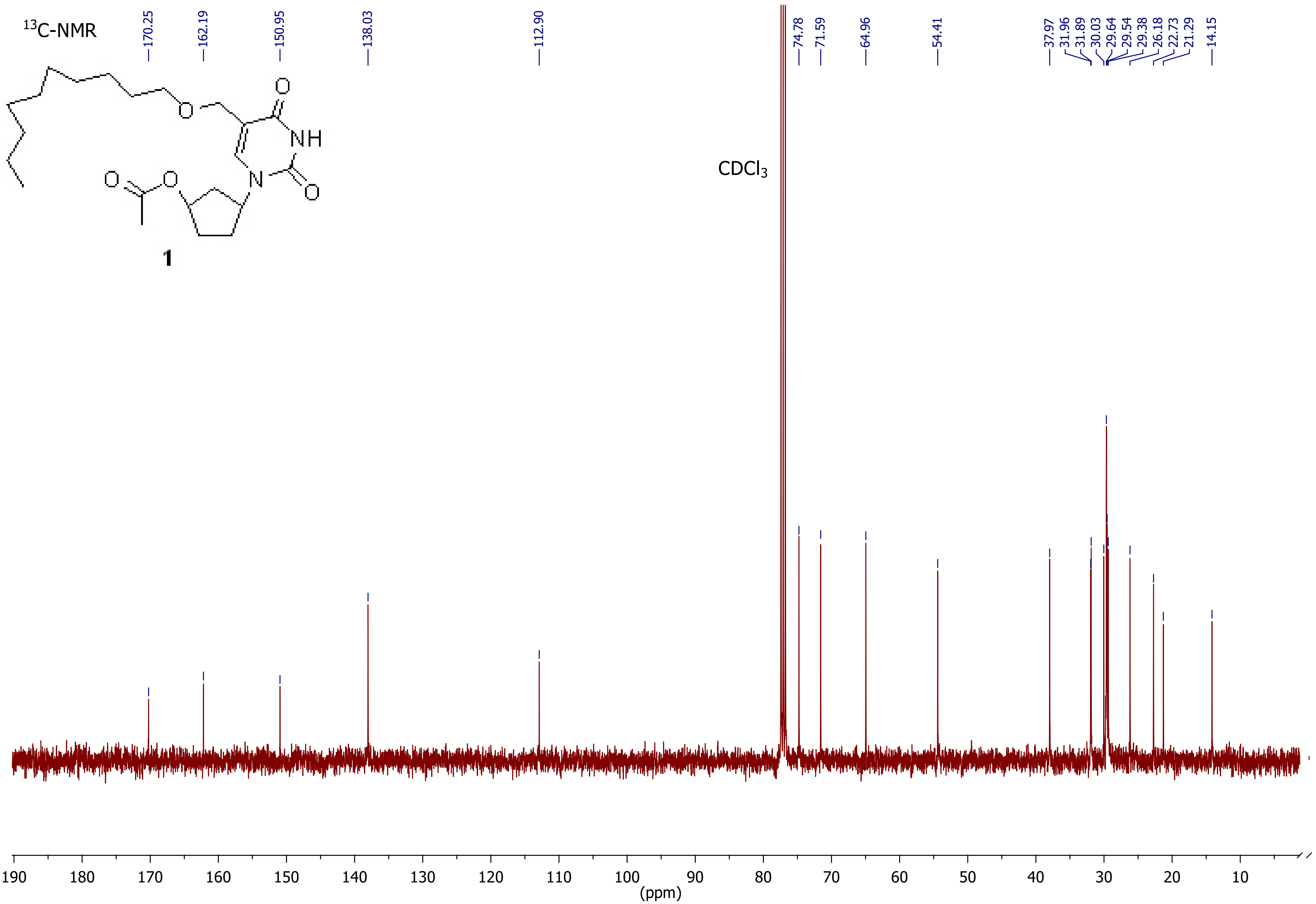

13C-NMR
170.25
162.19
150.95
138.03
112.90
74.78
71.59
64.96
54.41
37.97
31.96
31.89
30.03
29.64
29.54
29.38
26.18
22.73
21.29
14.15
O
NH
N
O
O
O
O
1
CDCl3
190
180
170
160
150
140
130
120
110
100
90
80
70
60
50
40
30
20
10
(ppm)

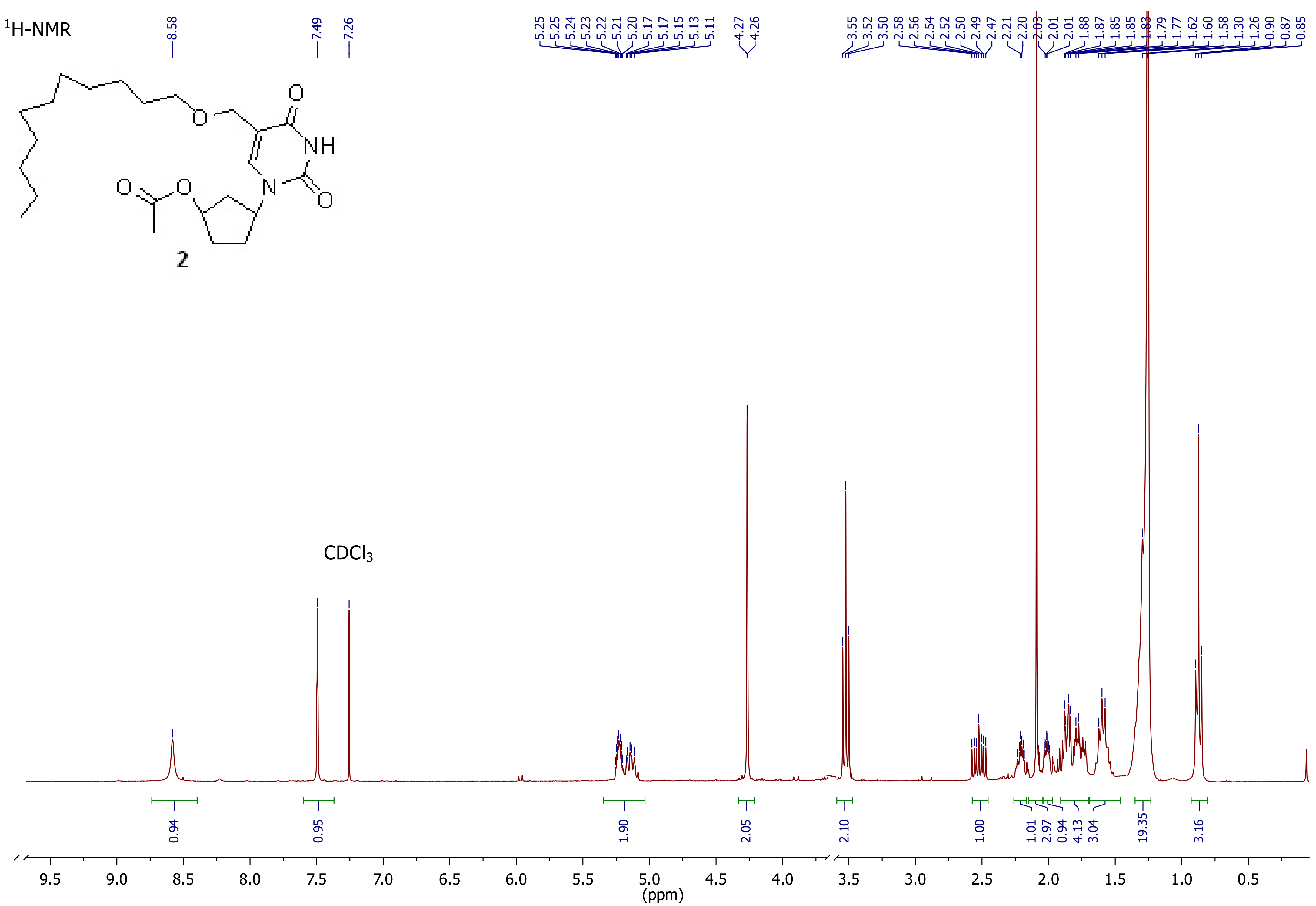

1H-NMR
8.58
7.49
7.26
5.25 5.25 5.24 5.23 5.22 5.21 5.20 5.17 5.17 5.15 5.13 5.11
4.27 4.26
3.55 3.52 3.50
2.58 2.56 2.54 2.52 2.50 2.49 2.47
2.21 2.20 2.03 2.01 2.01
1.88 1.87 1.85 1.85 1.83 1.79 1.77 1.62 1.60 1.58 1.30 1.26
0.90 0.87 0.85
O
NH
N
O
O
O
O
2
CDCl3
0.94 0.95 1.90 2.05 2.10 1.00 1.01 2.97 0.94 4.13 3.04 19.35 3.16
9.5 9.0 8.5 8.0 7.5 7.0 6.5 6.0 5.5 5.0 4.5 4.0 3.5 3.0 2.5 2.0 1.5 1.0 0.5
(ppm)

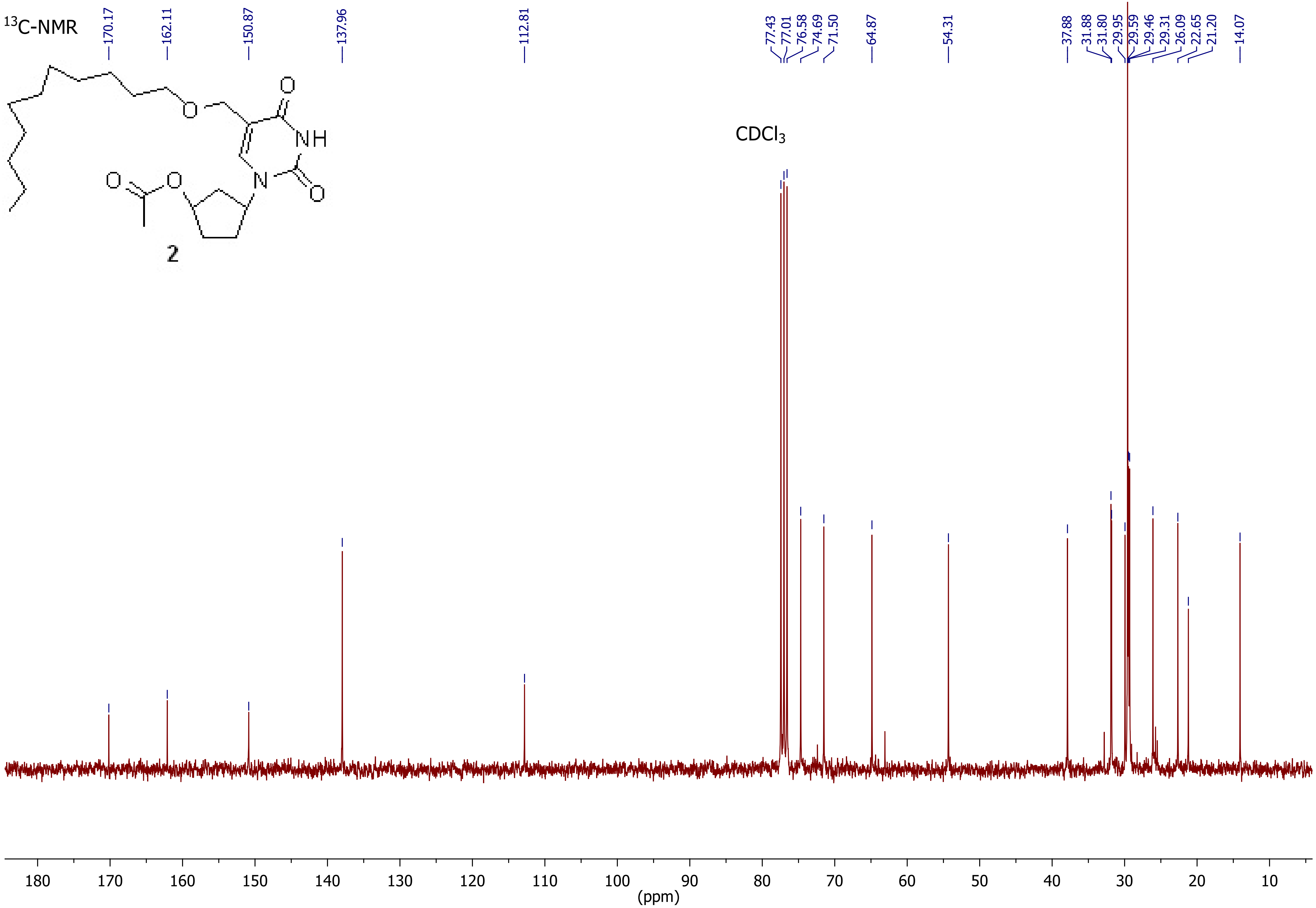

13C-NMR
170.17
162.11
150.87
137.96
112.81
77.43
77.01
76.58
74.69
71.50
64.87
54.31
37.88
31.88
31.80
29.95
29.59
29.46
29.31
26.09
22.65
21.20
14.07
O
NH
O
N
O
O
O
2
CDCl3
180 170 160 150 140 130 120 110 100 90 80 70 60 50 40 30 20 10
(ppm)

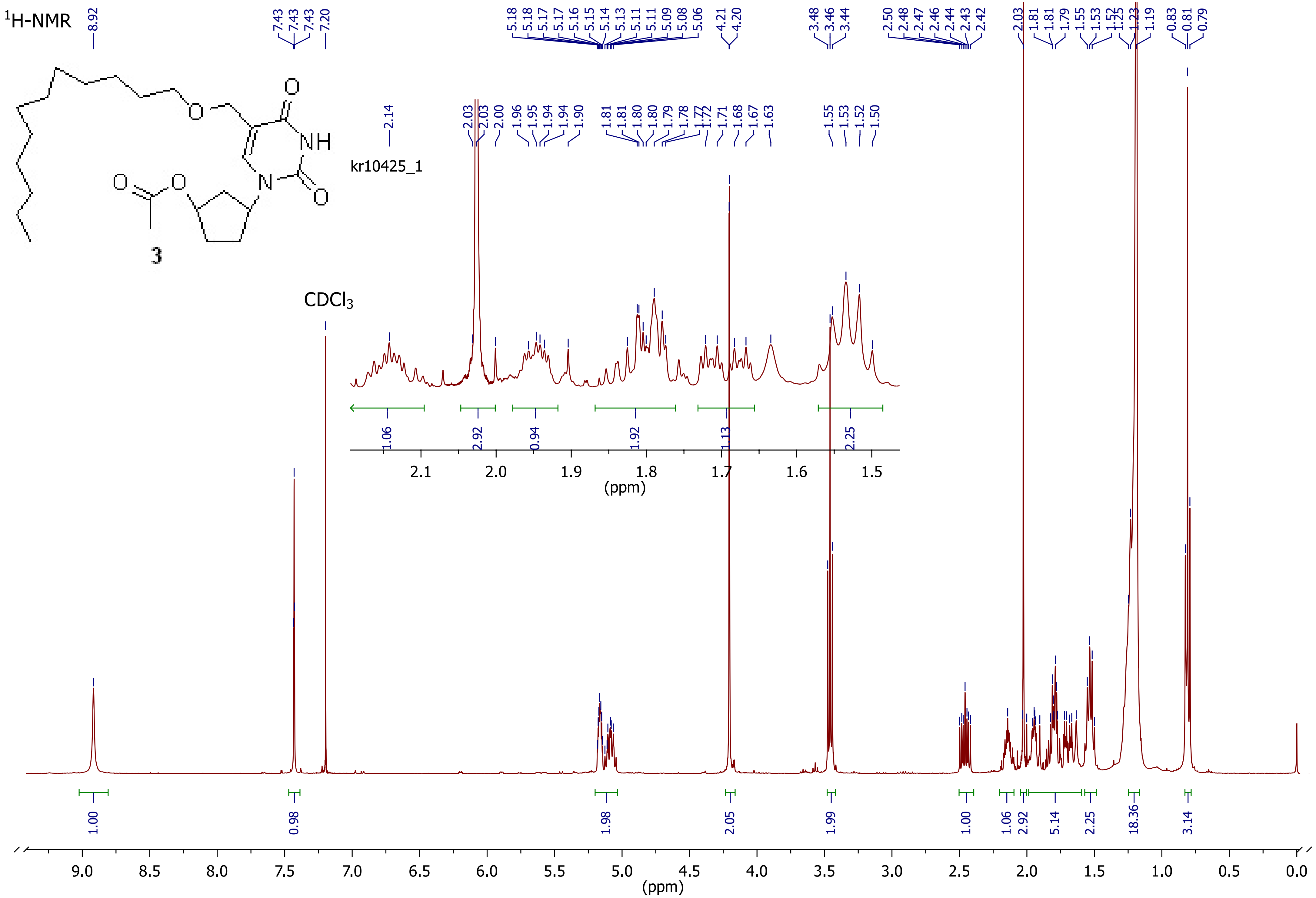

1H-NMR
8.92
7.43
7.43
7.43
7.20
5.18
5.18
5.17
5.17
5.16
5.15
5.14
5.13
5.11
5.11
5.09
5.08
5.06
4.21
4.20
3.48
3.46
3.44
2.50
2.48
2.47
2.46
2.44
2.43
2.42
2.03
1.81
1.81
1.79
1.55
1.53
1.52
1.25
1.23
1.19
0.83
0.81
0.79
O
O
NH
N
O
O
O
3
kr10425_1
2.14
2.03
2.03
2.00
1.96
1.95
1.94
1.94
1.90
1.81
1.81
1.80
1.80
1.79
1.78
1.77
1.72
1.71
1.68
1.67
1.63
1.55
1.53
1.52
1.50
CDCl3
1.06
2.92
0.94
1.92
1.13
2.25
2.1
2.0
1.9
1.8
1.7
1.6
1.5
(ppm)
1.00
0.98
1.98
2.05
1.99
1.00
1.06
2.92
5.14
2.25
18.36
3.14
9.0
8.5
8.0
7.5
7.0
6.5
6.0
5.5
5.0
4.5
4.0
3.5
3.0
2.5
2.0
1.5
1.0
0.5
0.0
(ppm)

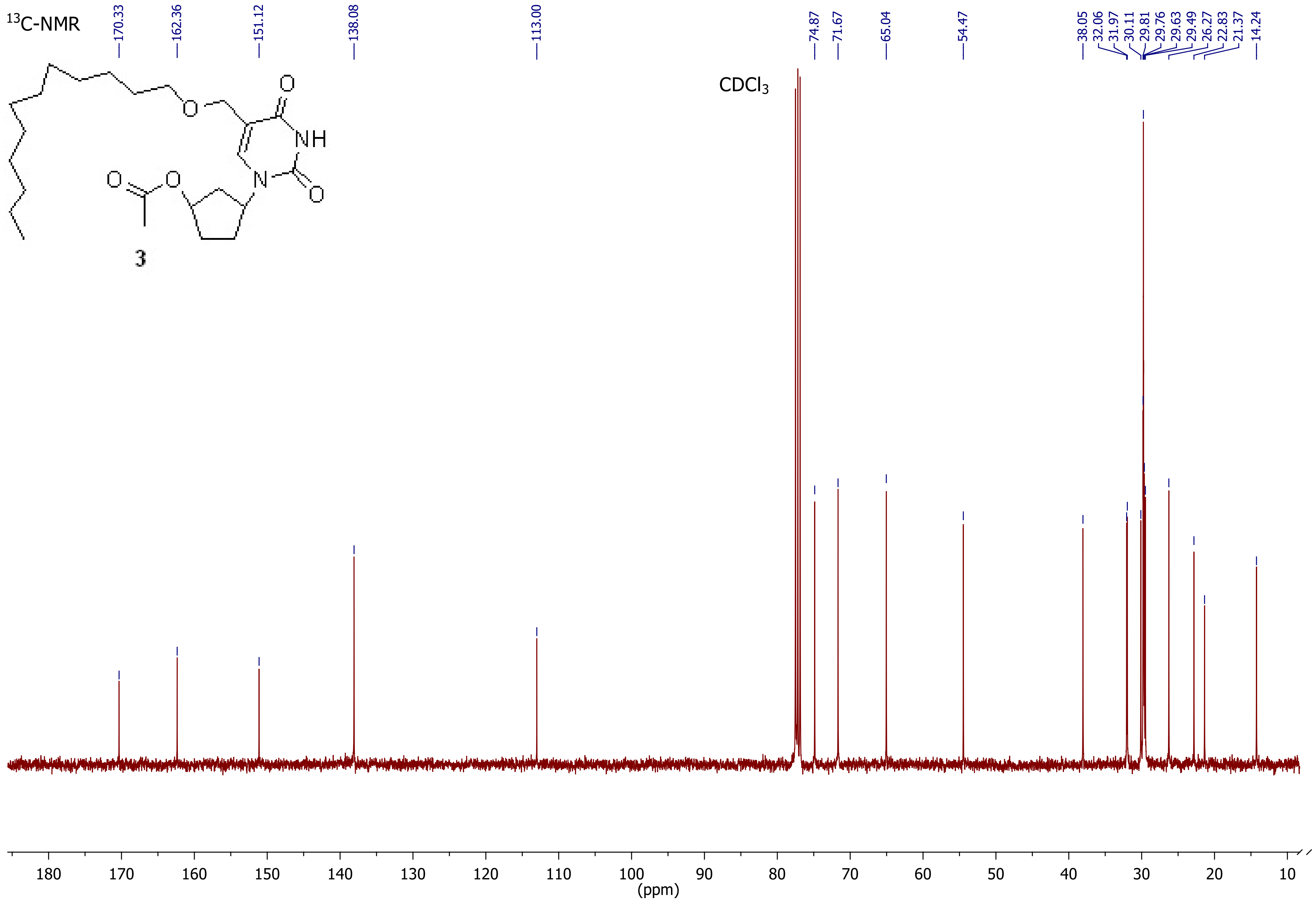

^{13}C-NMR
170.33
162.36
151.12
138.08
113.00
74.87
71.67
65.04
54.47
38.05
32.06
31.97
30.11
29.81
29.76
29.63
29.49
26.27
22.83
21.37
14.24
CDCl$_3$
O
NH
N
3
180 170 160 150 140 130 120 110 100 90 80 70 60 50 40 30 20 10
(ppm)

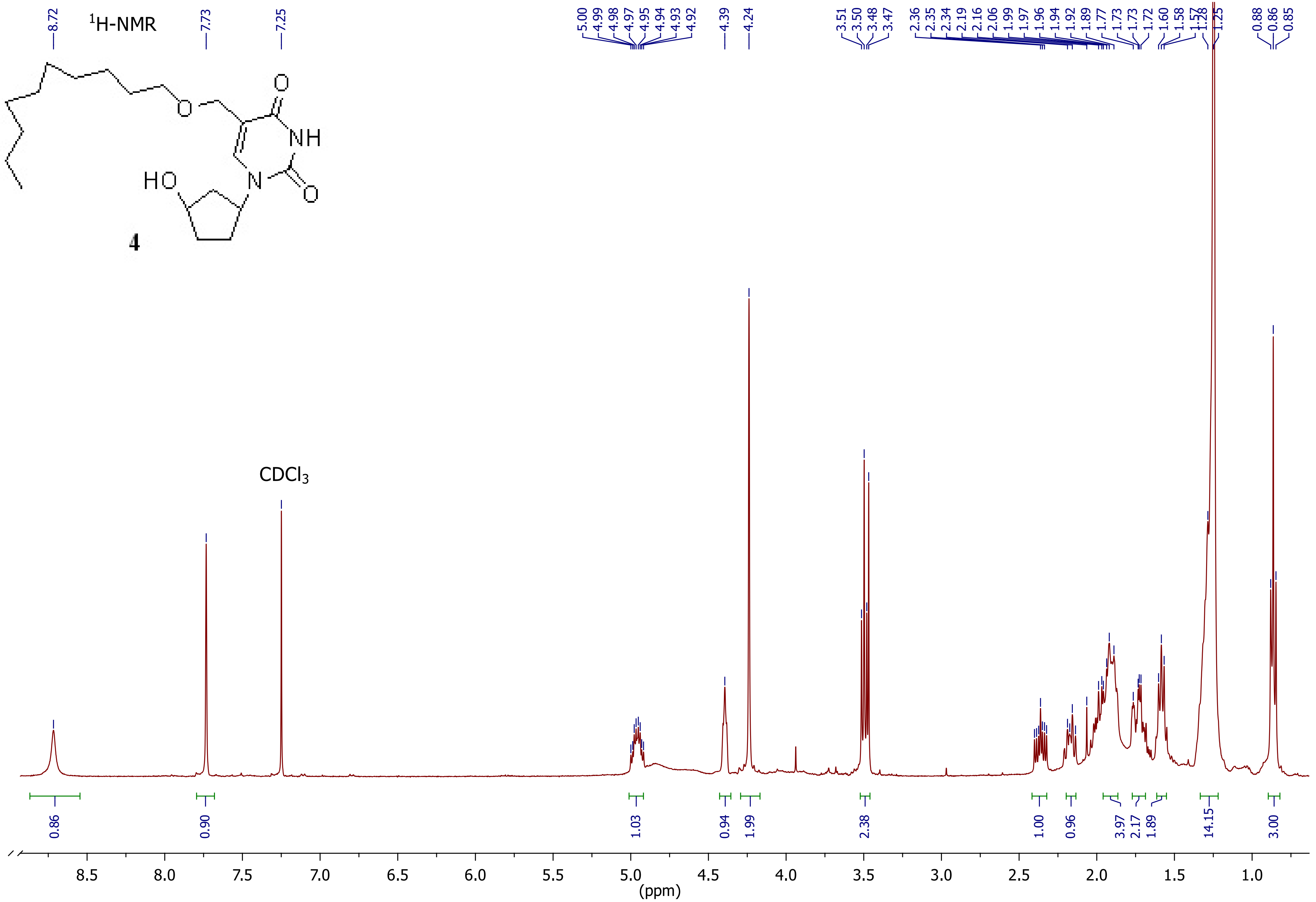

1H-NMR
4
CDCl3
8.72
7.73
7.25
5.00 4.99 4.98 4.97 4.95 4.94 4.93 4.92
4.39
4.24
3.51 3.50 3.48 3.47
2.36 2.35 2.34 2.19 2.16 2.06 1.99 1.97 1.96 1.94 1.92 1.89 1.77 1.73 1.73 1.72 1.60 1.58 1.57 1.28 1.25
0.88 0.86 0.85
0.86 0.90 1.03 0.94 1.99 2.38 1.00 0.96 3.97 2.17 1.89 14.15 3.00
8.5 8.0 7.5 7.0 6.5 6.0 5.5 5.0 4.5 4.0 3.5 3.0 2.5 2.0 1.5 1.0
(ppm)

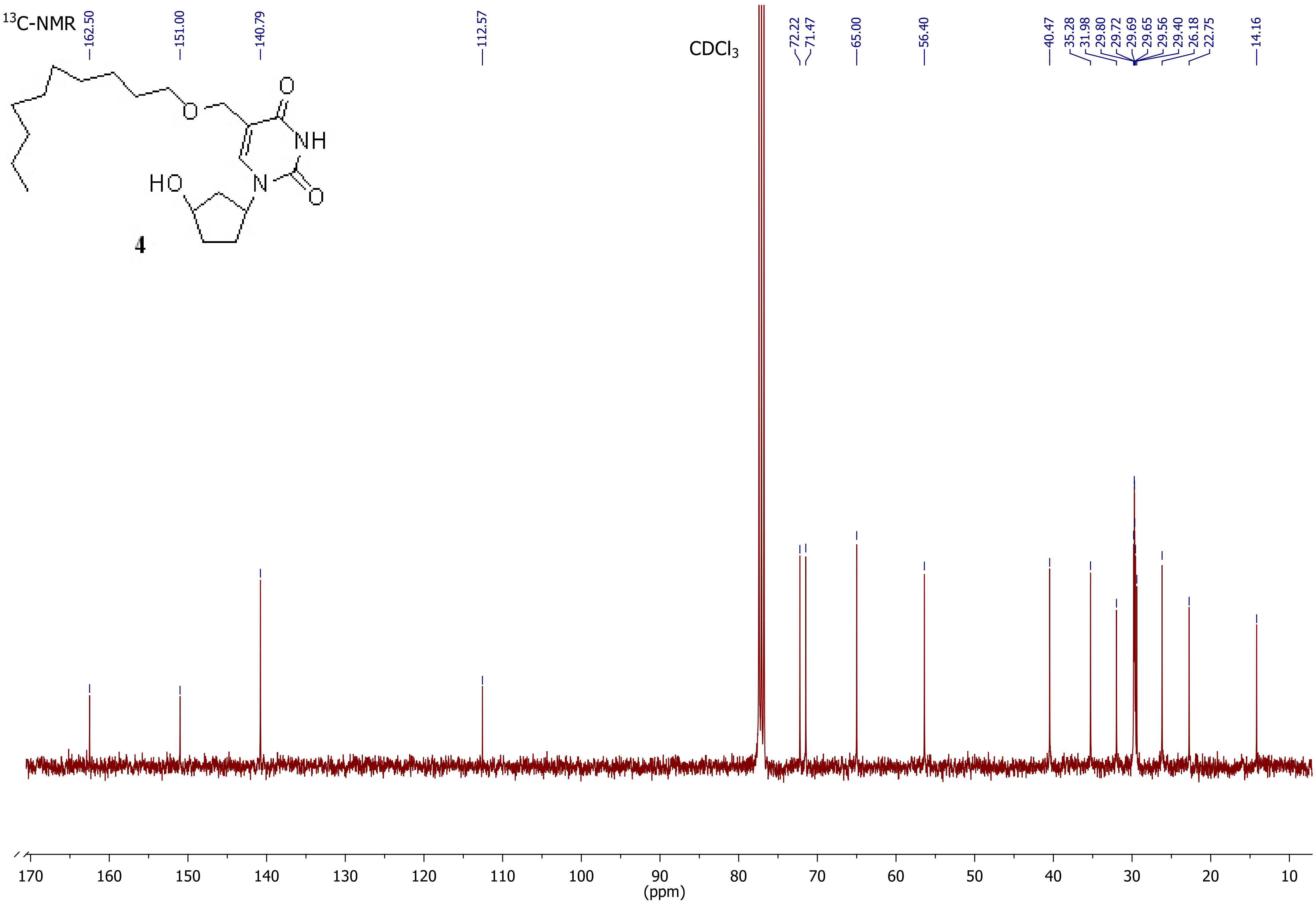

^{13}C-NMR
162.50
151.00
140.79
112.57
CDCl$_3$
72.22
71.47
65.00
56.40
40.47
35.28
31.98
29.80
29.72
29.69
29.65
29.56
29.40
26.18
22.75
14.16
O
NH
HO
N
O
O
4
170 160 150 140 130 120 110 100 90 80 70 60 50 40 30 20 10
(ppm)

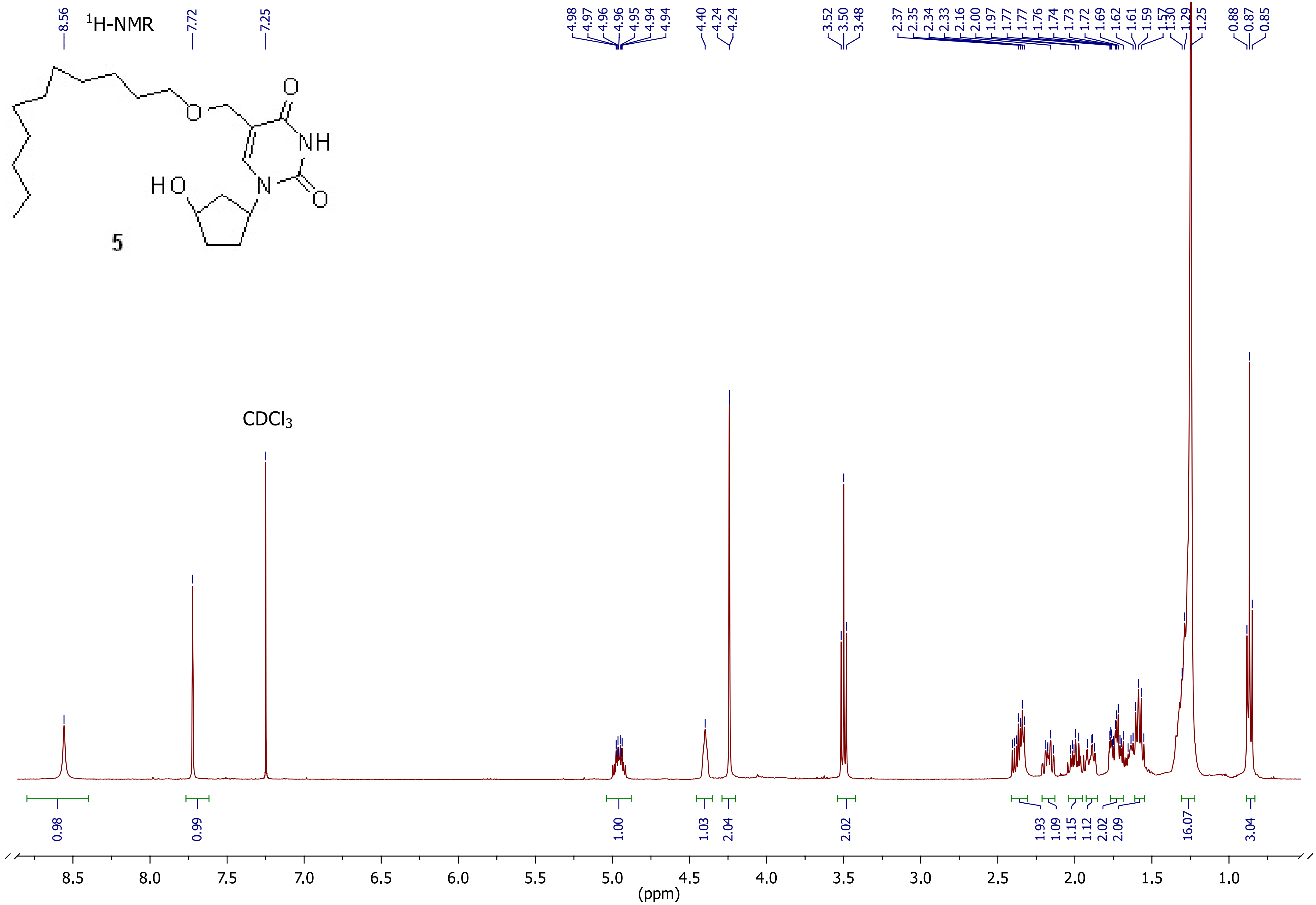

1H-NMR
5
HO
N
NH
O
CDCl3
8.56
7.72
7.25
4.98 4.97 4.96 4.96 4.95 4.94 4.94
4.40 4.24 4.24
3.52 3.50 3.48
2.37 2.35 2.34 2.33 2.16 2.00 1.97 1.77 1.77 1.76 1.74 1.73 1.72 1.69 1.62 1.61 1.59 1.57 1.30 1.29 1.25
0.88 0.87 0.85
0.98
0.99
1.00
1.03
2.04
2.02
1.93
1.09
1.15
1.12
2.02
2.09
16.07
3.04
8.5 8.0 7.5 7.0 6.5 6.0 5.5 5.0 4.5 4.0 3.5 3.0 2.5 2.0 1.5 1.0
(ppm)

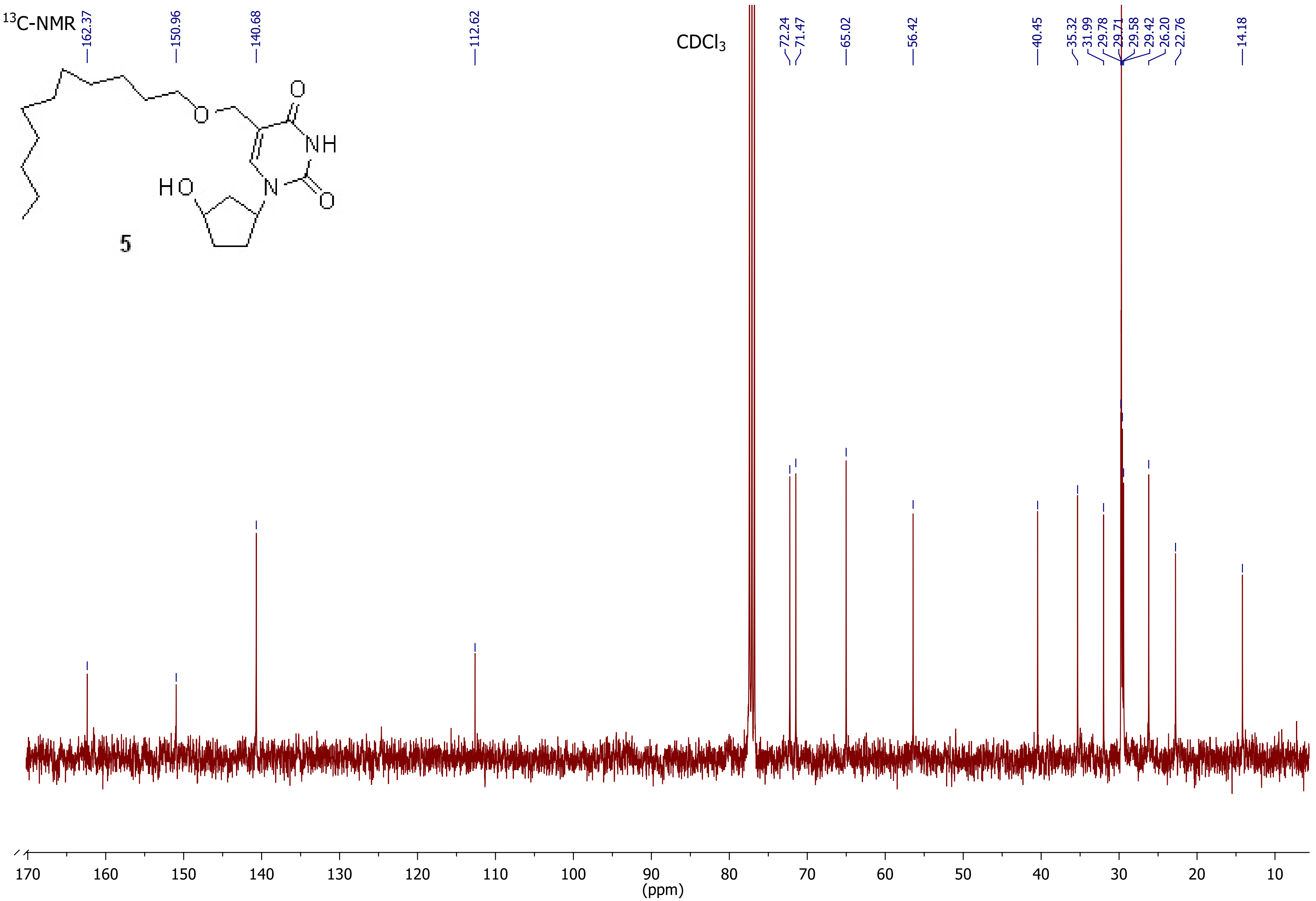

13C-NMR
162.37
150.96
140.68
112.62
CDCl3
72.24
71.47
65.02
56.42
40.45
35.32
31.99
29.78
29.71
29.58
29.42
26.20
22.76
14.18
O
NH
HO
N
O
O
5
170
160
150
140
130
120
110
100
90
80
70
60
50
40
30
20
10
(ppm)

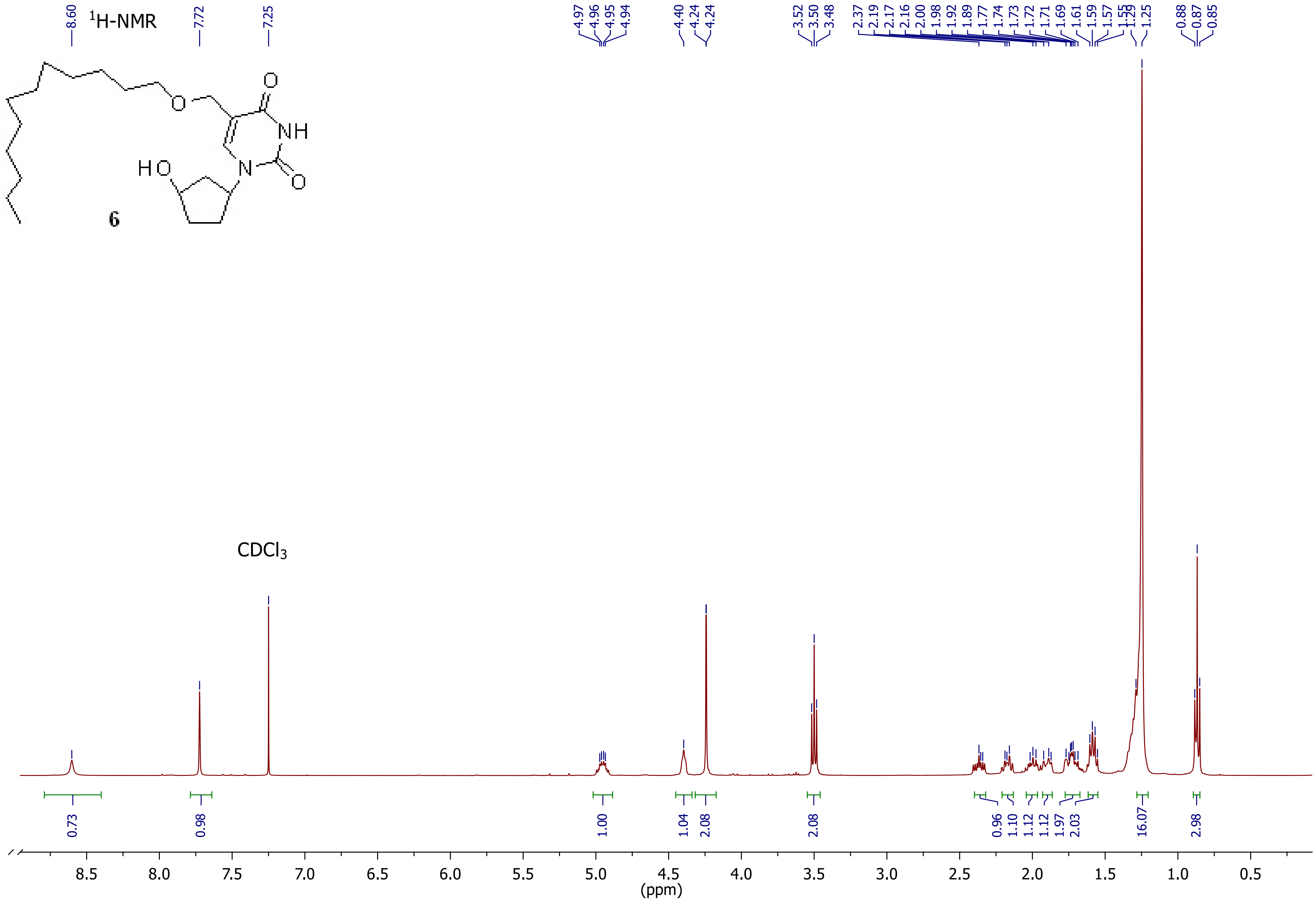

^{1}H-NMR
HO
N
NH
O
O
O
6
CDCl$_3$
8.60
7.72
7.25
4.97 4.96 4.95 4.94
4.40 4.24 4.24
3.52 3.50 3.48
2.37 2.19 2.17 2.16 2.00 1.98 1.92 1.89 1.77 1.74 1.73 1.72 1.71 1.69 1.61 1.59 1.57 1.55 1.29 1.25
0.88 0.87 0.85
0.73
0.98
1.00
1.04
2.08
2.08
0.96
1.10
1.12
1.12
1.97
2.03
16.07
2.98
8.5 8.0 7.5 7.0 6.5 6.0 5.5 5.0 4.5 4.0 3.5 3.0 2.5 2.0 1.5 1.0 0.5
(ppm)

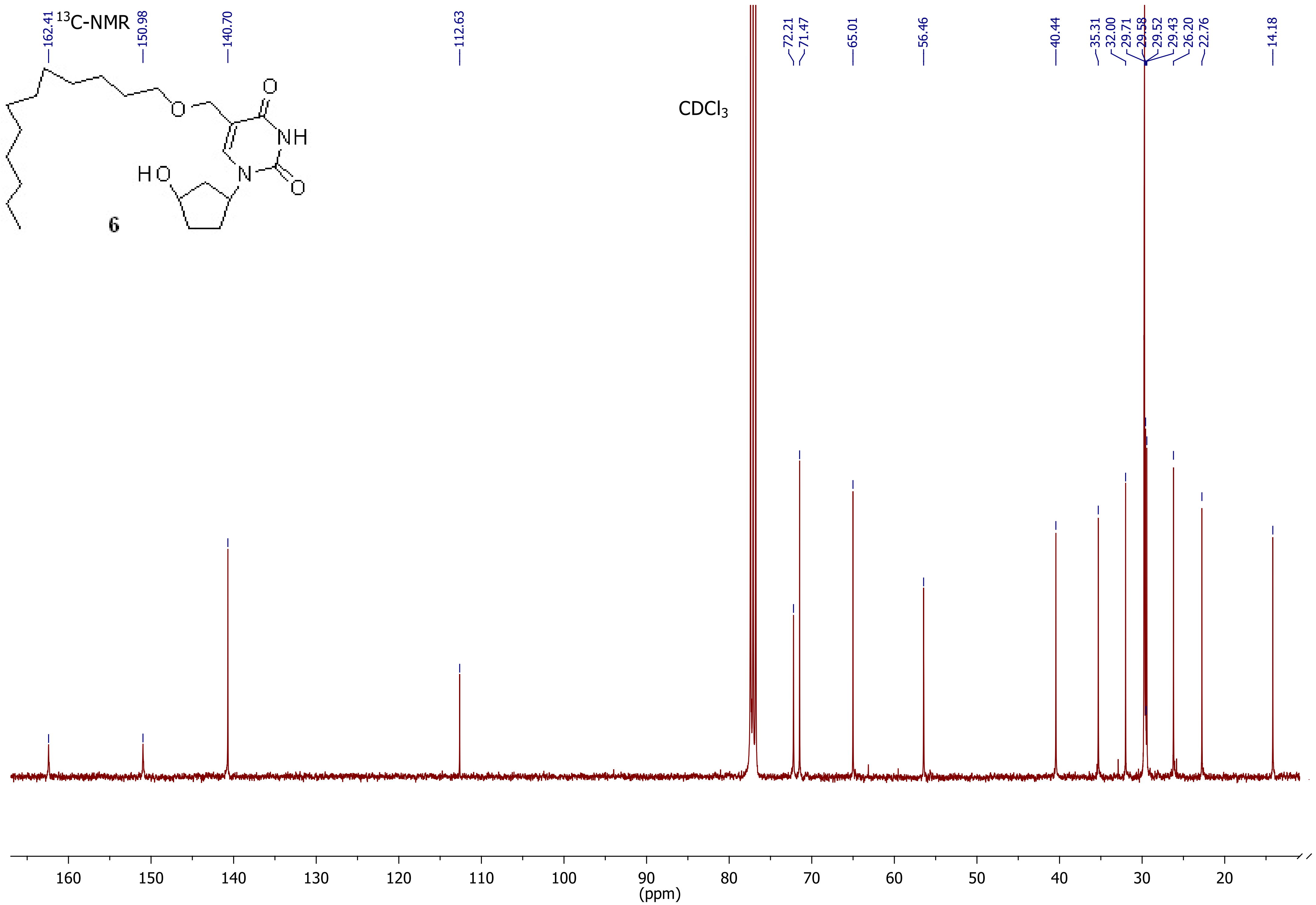

13C-NMR
162.41
150.98
140.70
112.63
72.21
71.47
65.01
56.46
40.44
35.31
32.00
29.71
29.58
29.52
29.43
26.20
22.76
14.18
CDCl3
O
NH
N
O
HO
O
6
160
150
140
130
120
110
100
90
80
70
60
50
40
30
20
(ppm)

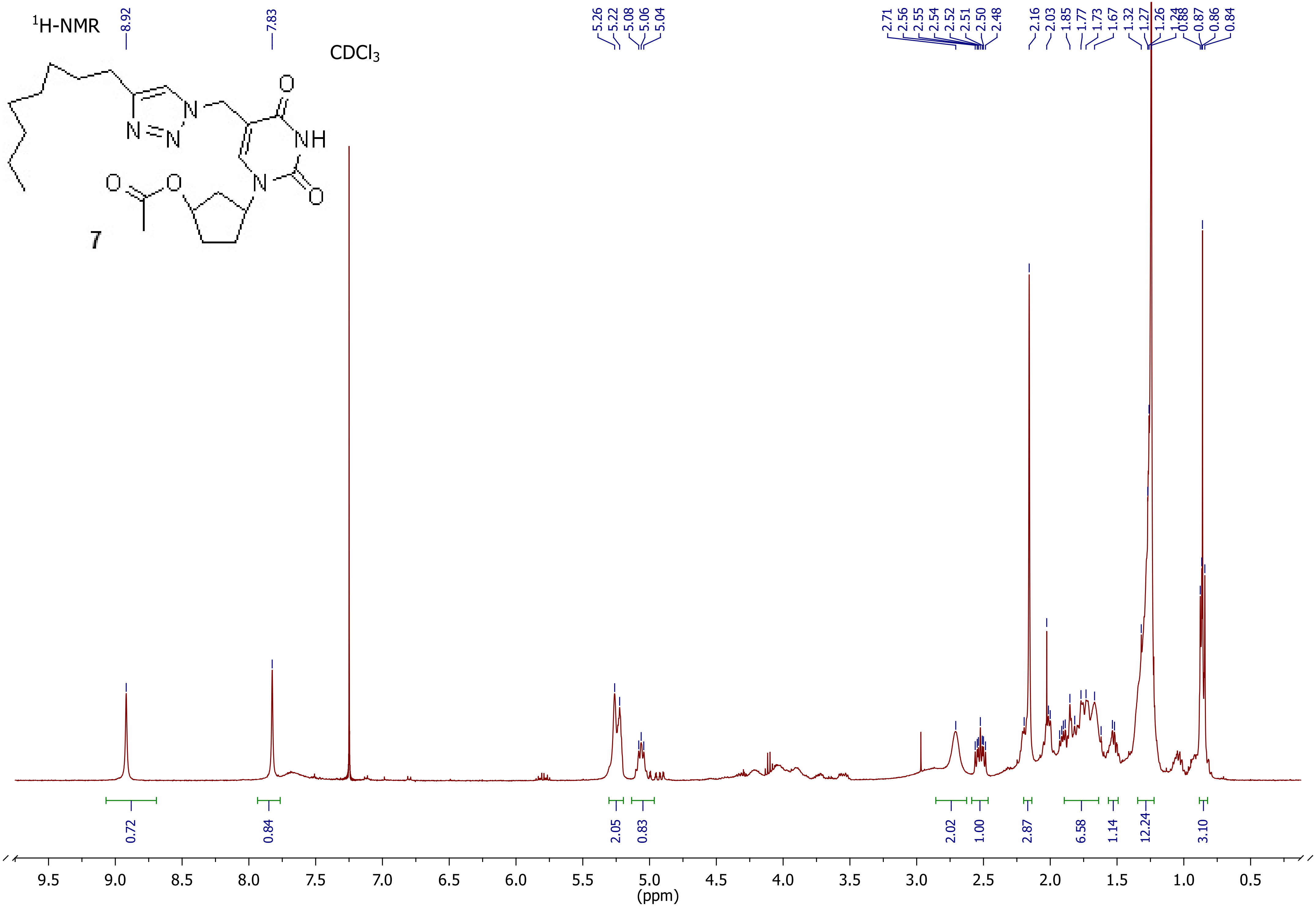

1H-NMR
CDCl3
7
8.92
7.83
5.26 5.22 5.08 5.06 5.04
2.71 2.56 2.55 2.54 2.52 2.51 2.50 2.48
2.16 2.03 1.85 1.77 1.73 1.67 1.32 1.27 1.26 1.24 0.88 0.87 0.86 0.84
0.72 0.84 2.05 0.83 2.02 1.00 2.87 6.58 1.14 12.24 3.10
9.5 9.0 8.5 8.0 7.5 7.0 6.5 6.0 5.5 5.0 4.5 4.0 3.5 3.0 2.5 2.0 1.5 1.0 0.5
(ppm)

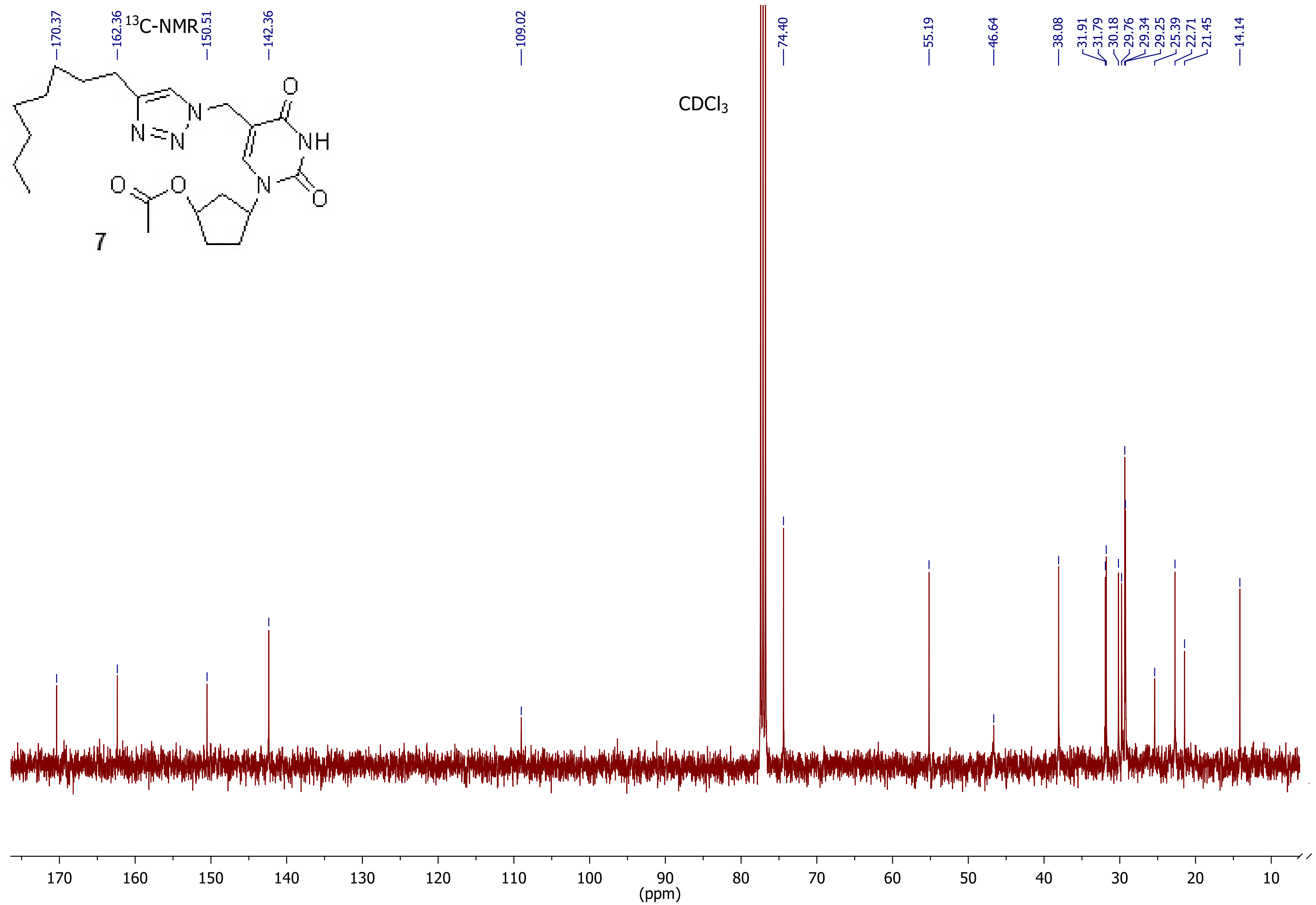

170.37
162.36
^{13}C-NMR
150.51
142.36
109.02
74.40
55.19
46.64
38.08
31.91
31.79
30.18
29.76
29.34
29.25
25.39
22.71
21.45
14.14
CDCl$_3$
7
(ppm)

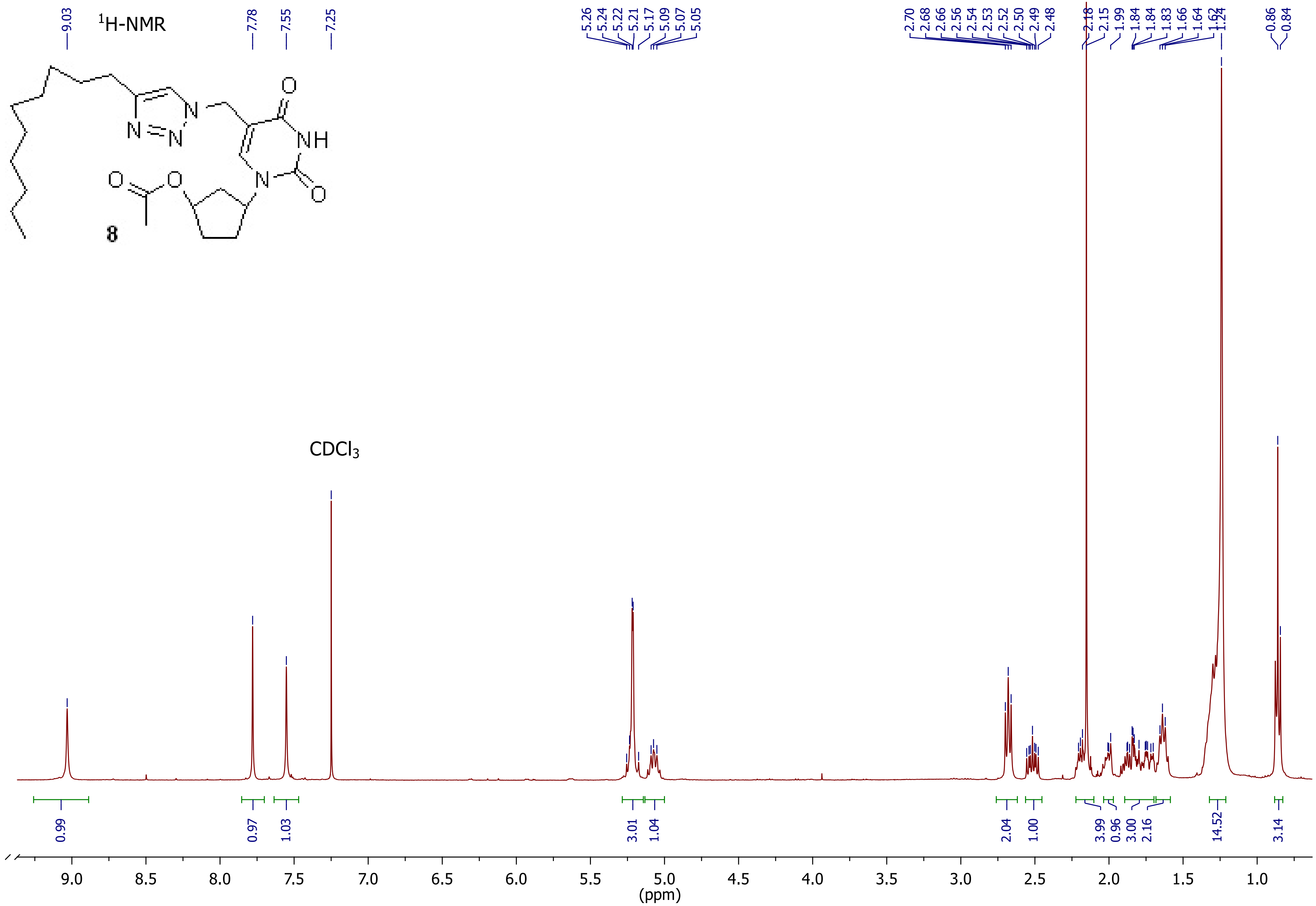

1H-NMR
8
CDCl3
9.03
7.78
7.55
7.25
5.26
5.24
5.22
5.21
5.17
5.09
5.07
5.05
2.70
2.68
2.66
2.56
2.54
2.53
2.52
2.50
2.49
2.48
2.18
2.15
1.99
1.84
1.84
1.83
1.66
1.64
1.62
1.24
0.86
0.84
0.99
0.97
1.03
3.01
1.04
2.04
1.00
3.99
0.96
3.00
2.16
14.52
3.14
9.0
8.5
8.0
7.5
7.0
6.5
6.0
5.5
5.0
4.5
4.0
3.5
3.0
2.5
2.0
1.5
1.0
(ppm)

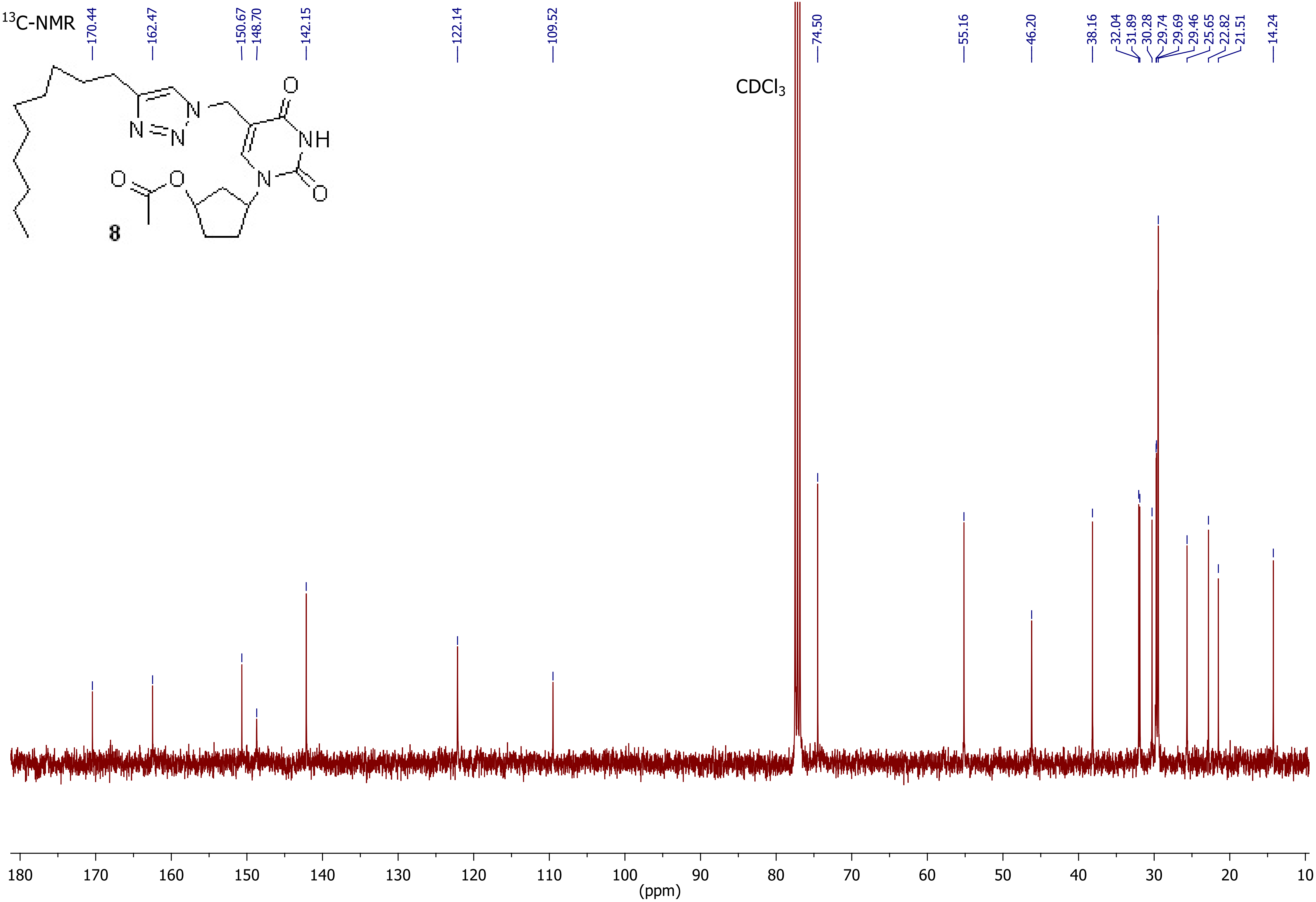

13C-NMR
170.44
162.47
150.67
148.70
142.15
122.14
109.52
74.50
55.16
46.20
38.16
32.04
31.89
30.28
29.74
29.69
29.46
25.65
22.82
21.51
14.24
CDCl3
NH
8
180 170 160 150 140 130 120 110 100 90 80 70 60 50 40 30 20 10
(ppm)

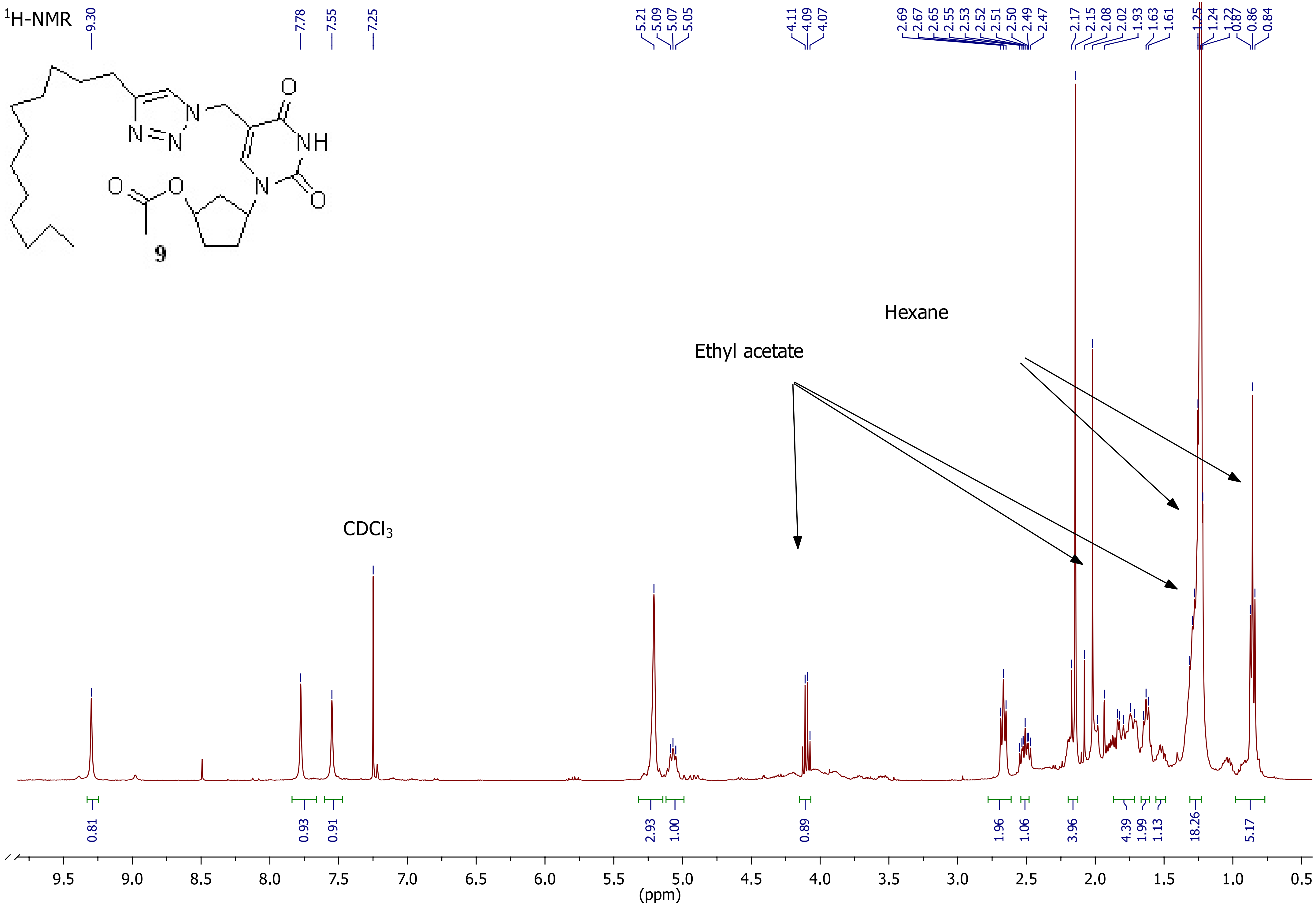

1H-NMR
9.30
7.78
7.55
7.25
5.21
5.09
5.07
5.05
4.11
4.09
4.07
2.69
2.67
2.65
2.55
2.53
2.52
2.51
2.50
2.49
2.47
2.17
2.15
2.08
2.02
1.93
1.63
1.61
1.25
1.24
1.22
0.87
0.86
0.84
O
N
N
N
NH
O
O
N
O
9
Hexane
Ethyl acetate
CDCl3
0.81
0.93
0.91
2.93
1.00
0.89
1.96
1.06
3.96
4.39
1.99
1.13
18.26
5.17
9.5
9.0
8.5
8.0
7.5
7.0
6.5
6.0
5.5
5.0
4.5
4.0
3.5
3.0
2.5
2.0
1.5
1.0
0.5
(ppm)

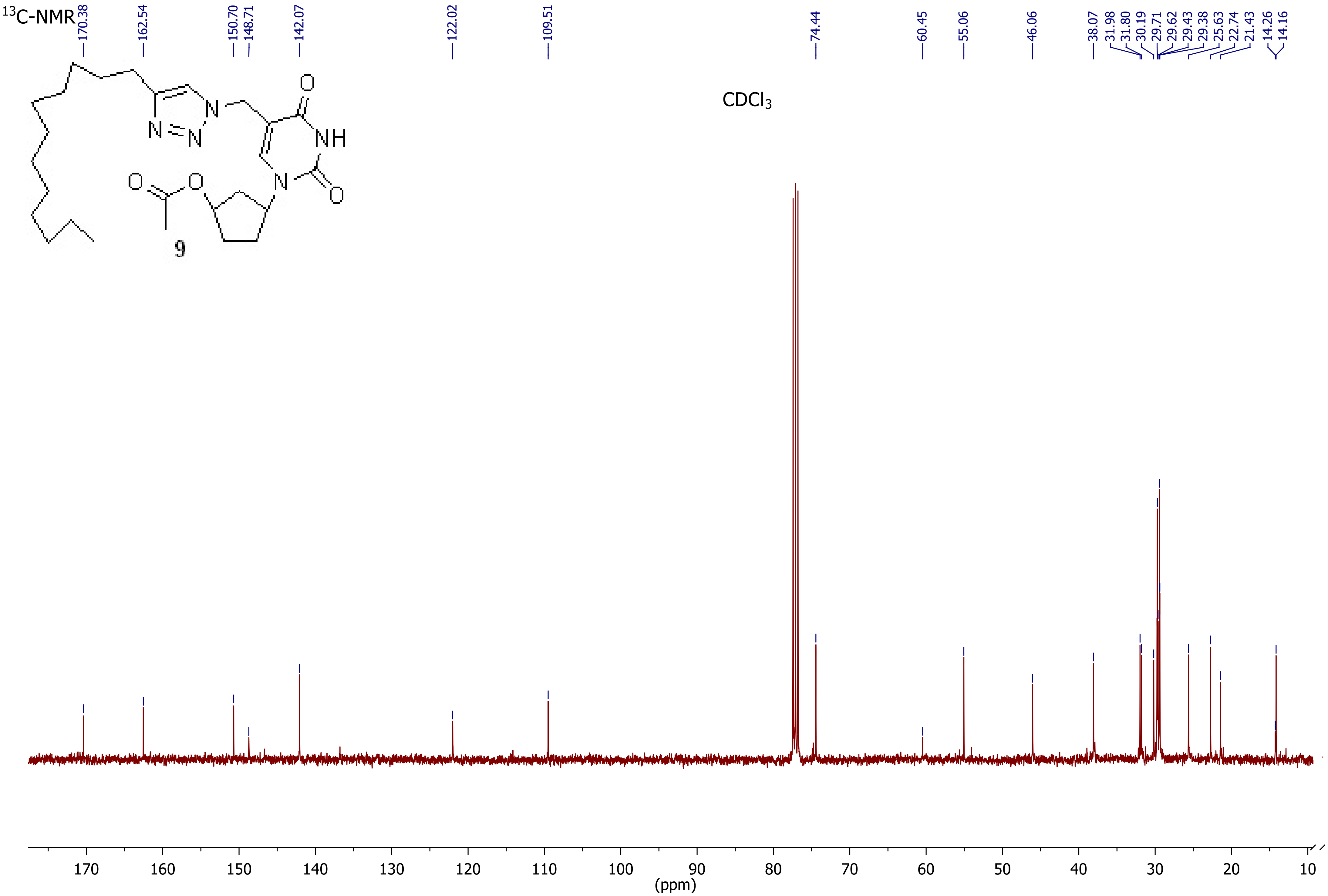

13C-NMR
170.38
162.54
150.70
148.71
142.07
122.02
109.51
74.44
60.45
55.06
46.06
38.07
31.98
31.80
30.19
29.71
29.62
29.43
29.38
25.63
22.74
21.43
14.26
14.16
CDCl3
O
N
NH
N
N
N
O
O
O
9
170
160
150
140
130
120
110
100
90
80
70
60
50
40
30
20
10
(ppm)

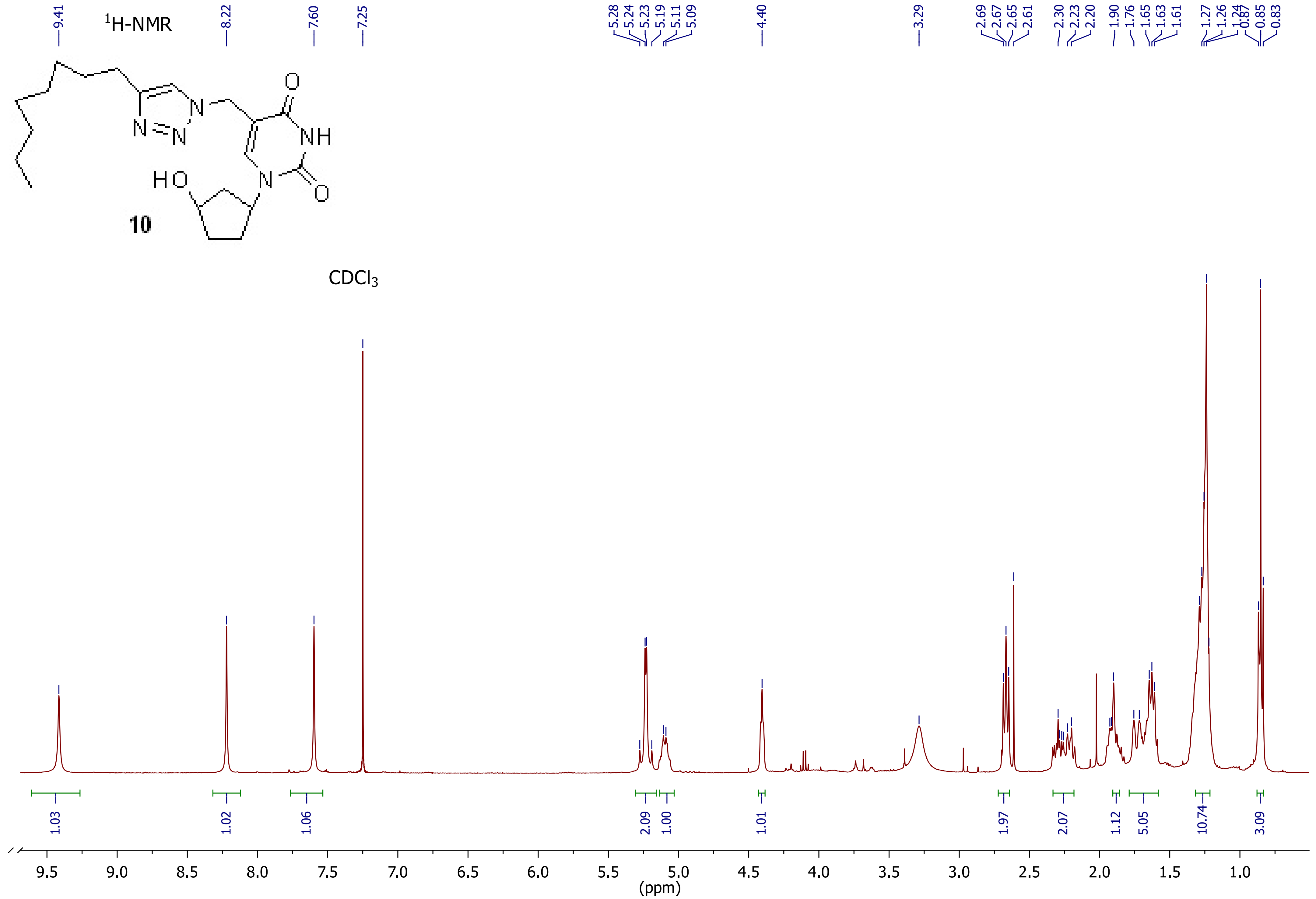

^{1}H-NMR
CDCl$_3$
9.41
8.22
7.60
7.25
5.28
5.24
5.23
5.19
5.11
5.09
4.40
3.29
2.69
2.67
2.65
2.61
2.30
2.23
2.20
1.90
1.76
1.65
1.63
1.61
1.27
1.26
1.24
0.87
0.85
0.83
1.03
1.02
1.06
2.09
1.00
1.01
1.97
2.07
1.12
5.05
10.74
3.09
9.5
9.0
8.5
8.0
7.5
7.0
6.5
6.0
5.5
5.0
4.5
4.0
3.5
3.0
2.5
2.0
1.5
1.0
(ppm)

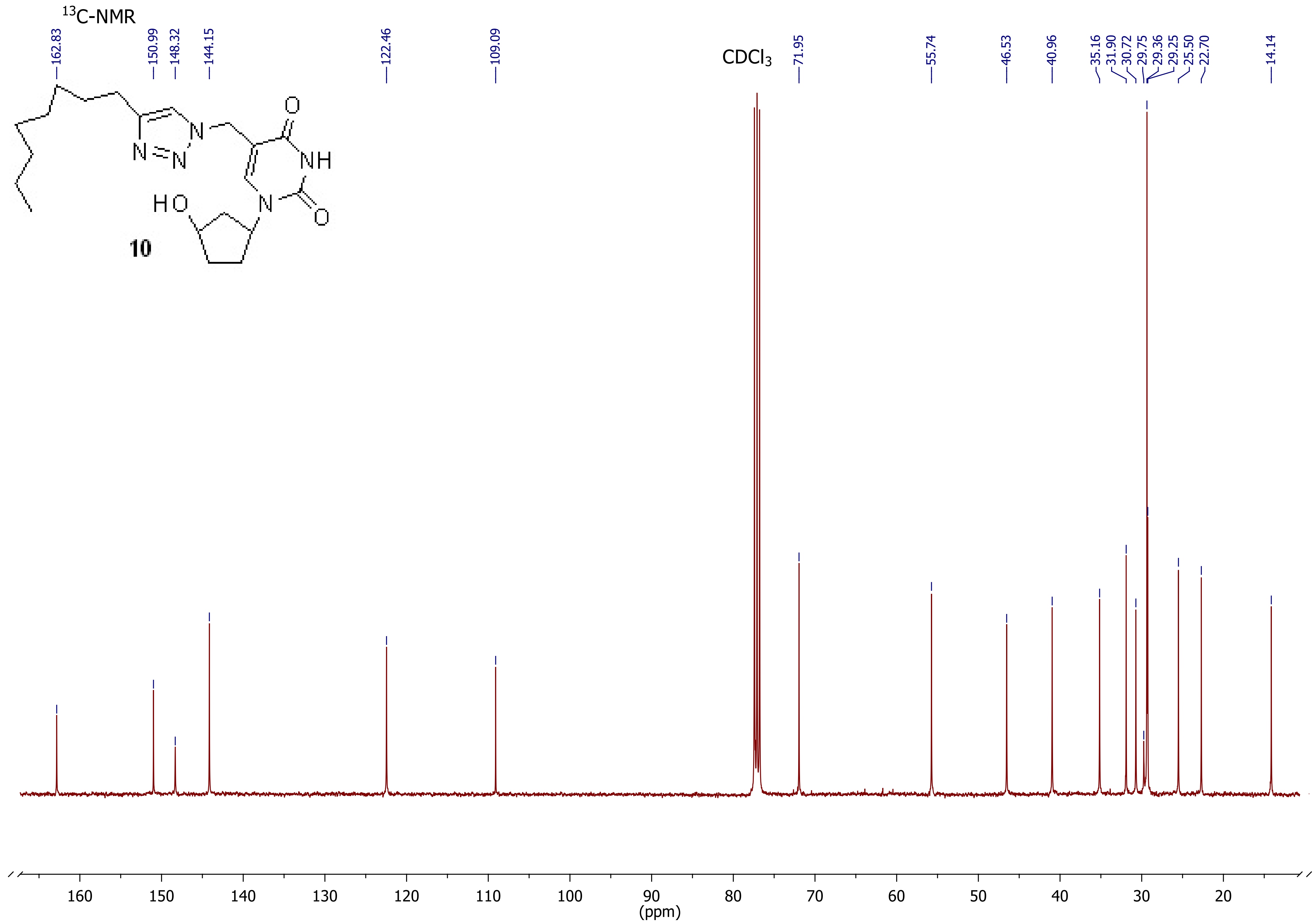

13C-NMR
162.83
150.99
148.32
144.15
122.46
109.09
CDCl3
71.95
55.74
46.53
40.96
35.16
31.90
30.72
29.75
29.36
29.25
25.50
22.70
14.14
10
(ppm)

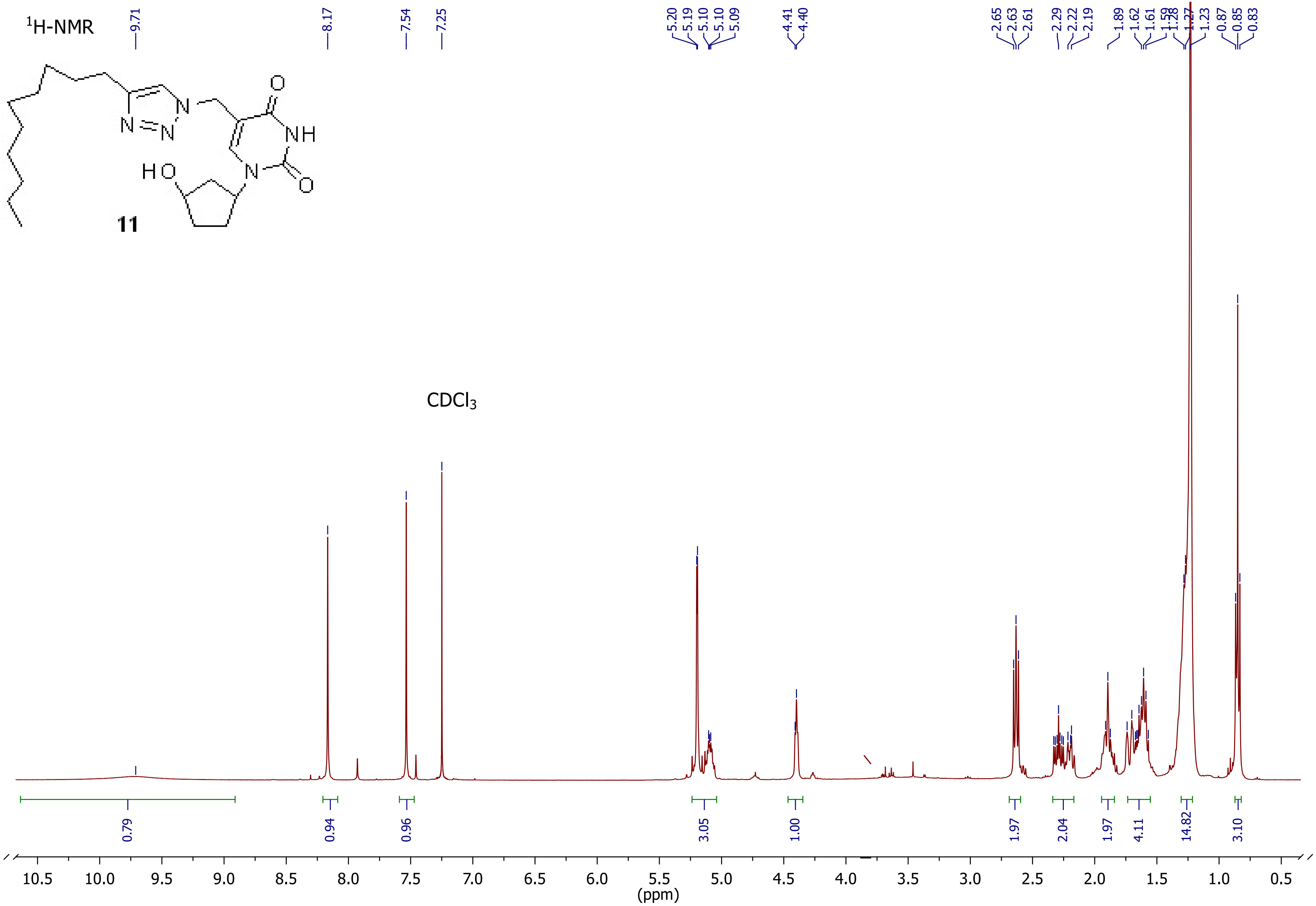

^{1}H-NMR
11
CDCl3
9.71
8.17
7.54
7.25
5.20
5.19
5.10
5.10
5.09
4.41
4.40
2.65
2.63
2.61
2.29
2.22
2.19
1.89
1.62
1.61
1.59
1.28
1.27
1.23
0.87
0.85
0.83
0.79
0.94
0.96
3.05
1.00
1.97
2.04
1.97
4.11
14.82
3.10
10.5
10.0
9.5
9.0
8.5
8.0
7.5
7.0
6.5
6.0
5.5
5.0
4.5
4.0
3.5
3.0
2.5
2.0
1.5
1.0
0.5
(ppm)

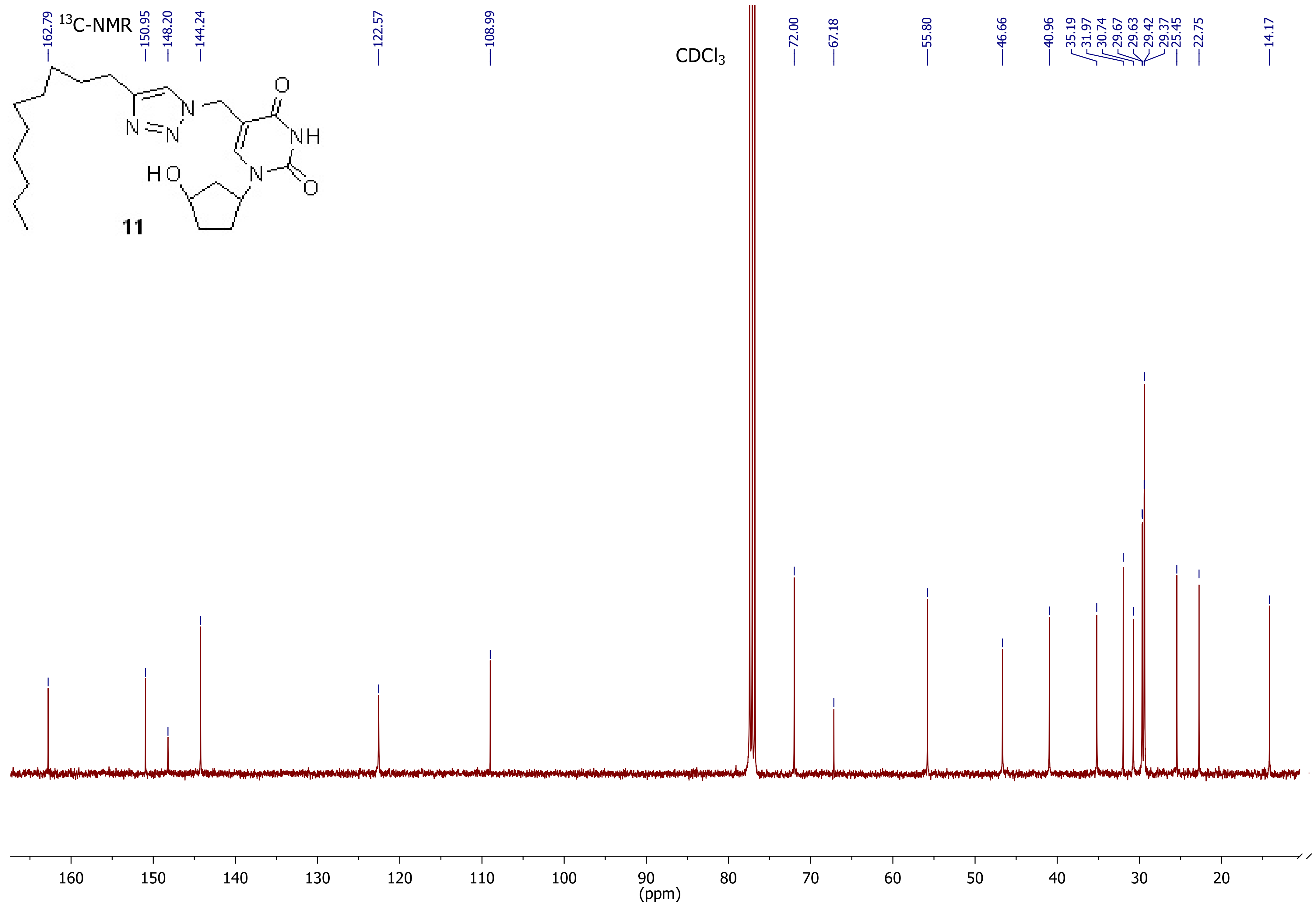

13C-NMR
162.79
150.95
148.20
144.24
122.57
108.99
CDCl3
72.00
67.18
55.80
46.66
40.96
35.19
31.97
30.74
29.67
29.63
29.42
29.37
25.45
22.75
14.17
O
N
N
N
NH
HO
N
O
11
160
150
140
130
120
110
100
90
80
70
60
50
40
30
20
(ppm)

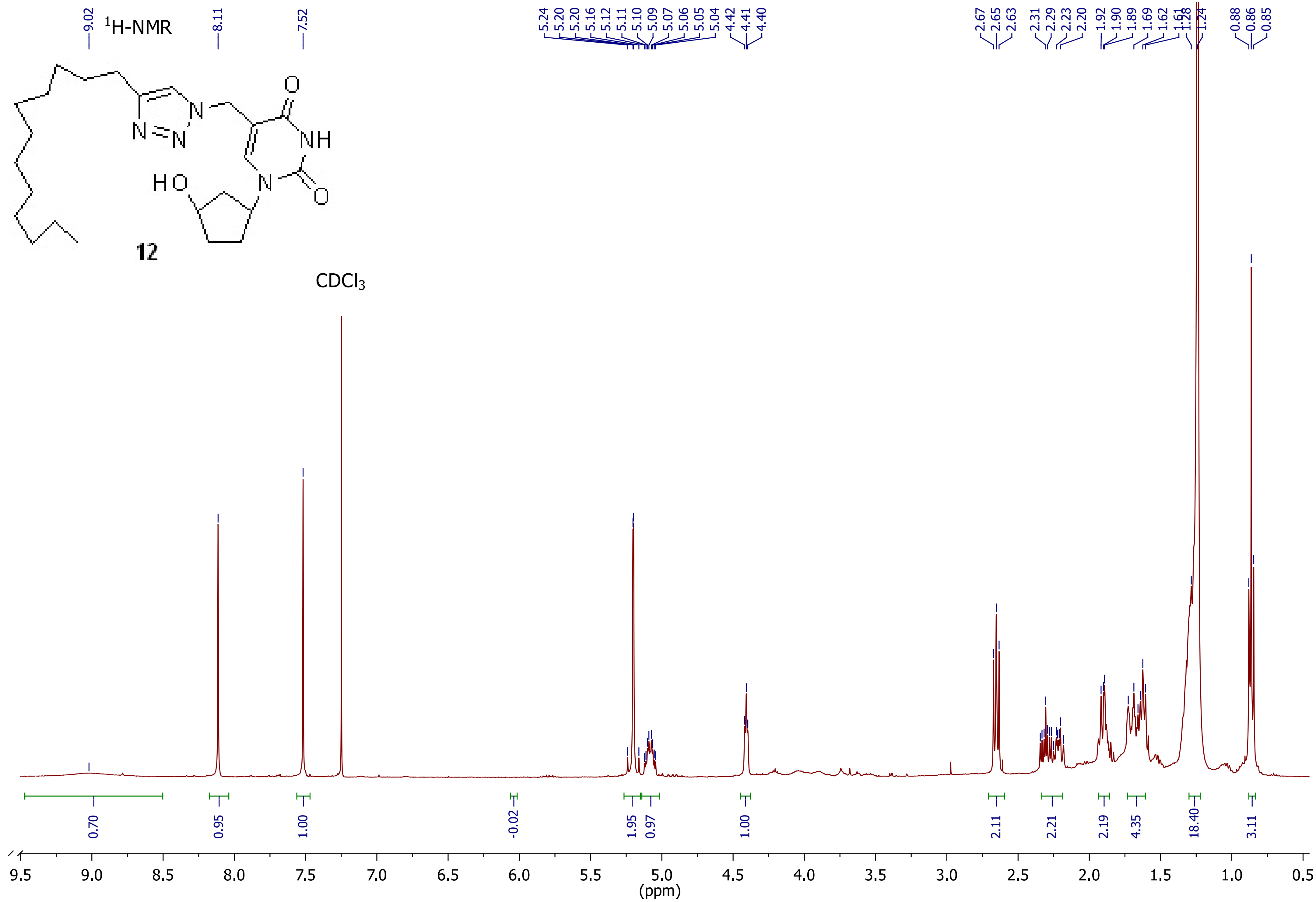

1H-NMR
9.02
8.11
7.52
5.24 5.20 5.20 5.16 5.12 5.11 5.10 5.09 5.07 5.06 5.05 5.04 4.42 4.41 4.40
2.67 2.65 2.63 2.31 2.29 2.23 2.20 1.92 1.90 1.89 1.69 1.62 1.61 1.28 1.24 0.88 0.86 0.85
N
N
N
O
NH
N
O
HO
12
CDCl3
0.70
0.95
1.00
-0.02
1.95
0.97
1.00
2.11
2.21
2.19
4.35
18.40
3.11
9.5 9.0 8.5 8.0 7.5 7.0 6.5 6.0 5.5 5.0 4.5 4.0 3.5 3.0 2.5 2.0 1.5 1.0 0.5
(ppm)

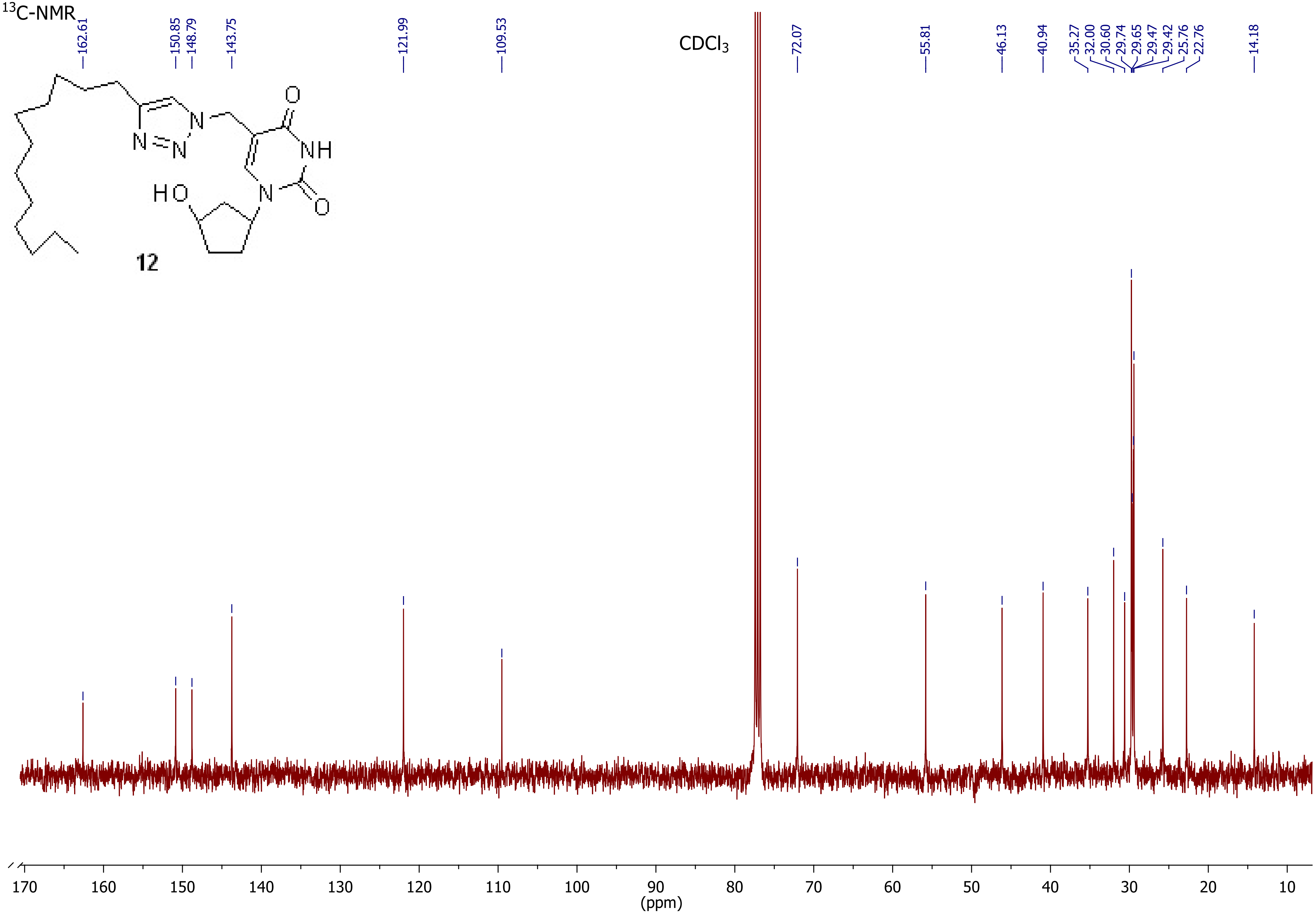
^{13}C-NMR
162.61
150.85
148.79
143.75
121.99
109.53
$CDCl_3$
72.07
55.81
46.13
40.94
35.27
32.00
30.60
29.74
29.65
29.47
29.42
25.76
22.76
14.18
O
N
N
N
NH
HO
N
O
12
170 160 150 140 130 120 110 100 90 80 70 60 50 40 30 20 10
(ppm)

Table . The antimycobacterial activity of 5'-norcarbocyclic derivatives of 5-substituted uracil

Compounds	Concentration, μg/ml	*M.tuberculosis* H37Rv			*M.tuberculosis* MS-115		
		Culture growth initiation, days (Mean±SD)	Growth inhibition (%)	MIC(99), μg/ml	Culture growth initiation, days (Mean±SD)	Growth inhibition (%)	MIC(99), μg/ml
drug-free control		6,00±0,12	-	-	5,66±0,02	-	-
INH	0,1	No culture growth	100	≤0,1	5,80±0,02	No	>100
RIF	1	No culture growth	100	≤1	5,73±0,08	No	>50
LFX	1,5	No culture growth	100	≤1,5	No culture growth	100	≤1,5
**(±)3**	2,5	6,02±0,25	No	20	5,72±0,09	No	20
	5	7,37±0,64	No		5,95±0,11	No	
	10	9,80±0,40	90		7,74±0,34	75	
	20	11,05±3,54	99		12,23[1)]	99	
	50	8,57±0,27[1)]	99		No culture growth	100	
**(±)7**	2,5	5,82±0,09	No	50	5,62±0,06	No	20
	5	6,14±0,05	No		5,84±0,10	No	
	10	8,11±0,23	75		6,48±0,11	No	
	20	9,31±0,31	90		12,35±2,00[1)]	99	
	50	10,38[1)]	99		No culture growth	100	
**(±)8**	2,5	5,17±0,04	No	50	5,65±0,15	No	50
	5	5,27±0,05	No		5,58±0,09	No	
	10	5,81±0,06	No		5,85±0,11	No	
	20	7,73±0,27	No		6,84±0,27	No	
	50	No culture growth	100		No culture growth	100	

[1)] growth in one sample from three
